# Partial Unwrapping and Histone Tail Dynamics in Nucleosome Revealed by Coarse-Grained Molecular Simulations

**DOI:** 10.1371/journal.pcbi.1004443

**Published:** 2015-08-11

**Authors:** Hiroo Kenzaki, Shoji Takada

**Affiliations:** 1 Advanced Center for Computing and Communication, RIKEN, Hirosawa, Wako, Saitama, Japan; 2 Department of Biophysics, Graduate School of Science, Kyoto University, Kyoto, Kitashirakawa Sakyo, Kyoto, Japan; Ottawa University, CANADA

## Abstract

Nucleosomes, basic units of chromatin, are known to show spontaneous DNA unwrapping dynamics that are crucial for transcriptional activation, but its structural details are yet to be elucidated. Here, employing a coarse-grained molecular model that captures residue-level structural details up to histone tails, we simulated equilibrium fluctuations and forced unwrapping of single nucleosomes at various conditions. The equilibrium simulations showed spontaneous unwrapping from outer DNA and subsequent rewrapping dynamics, which are in good agreement with experiments. We found several distinct partially unwrapped states of nucleosomes, as well as reversible transitions among these states. At a low salt concentration, histone tails tend to sit in the concave cleft between the histone octamer and DNA, tightening the nucleosome. At a higher salt concentration, the tails tend to bound to the outer side of DNA or be expanded outwards, which led to higher degree of unwrapping. Of the four types of histone tails, H3 and H2B tail dynamics are markedly correlated with partial unwrapping of DNA, and, moreover, their contributions were distinct. Acetylation in histone tails was simply mimicked by changing their charges, which enhanced the unwrapping, especially markedly for H3 and H2B tails.

## Author Summary

Nucleosomes, folding units of chromatin, wrap DNA about 1.75 turns and provide bottlenecks for transcription. Recent experiments showed that nucleosomes are not rigid but dynamic, showing spontaneous and partial unwrapping which is thus important for transcriptional activation. Experimentally, however, one cannot directly watch DNA unwrapping at high resolution. On the other hand, molecular dynamics simulations have high spatio-temporal resolution and thus can be powerful and complementary to experiments. Here, we put forward coarse-grained modeling of protein-DNA interactions at residue-level resolution, which is rather generic and thus can be applied to any protein-DNA complexes. By this method, we could reveal spontaneous and salt-concentration dependent partial unwrapping of DNA from nucleosomes. In addition to consistency with single molecule experiments, the simulation showed multiple and distinct intermediate states of unwrapping. Interestingly, partial unwrapping of DNA is correlated with certain parts of histone tail dynamics. Deleting positive charges in histone tails that mimics histone acetylation facilitated partial unwrapping, most significantly for H3 and H2B.

## Introduction

Nucleosomes, basic units of chromatin, are made of about 147 base pair (bp) double strand (ds) DNA wrapped 1.75 turns around a histone octamer [1]. Many available X-ray crystal structures provide atomic structural information on nucleosomes [2–7]. Yet, nucleosomes are not static, but dynamic complexes changing their structures, positions along genome, and component during cell cycles. Upon DNA replication, for example, nucleosomes must globally disassemble and, after replication, re-assemble, which also involves nucleosome repositioning [8,9]. In addition to these global changes, nucleosomes regularly show partial unwrapping dynamics, which could control higher-order chromatin folding and transcriptional activity [10,11]. These dynamic aspects of nucleosomes are much less clear.

Recently, partial and global unwrapping of single nucleosomes have been intensively investigated by single-molecule FRET experiments, mechanical pulling experiments with optical traps, and so forth [12–24]. Notably, single-molecule FRET experiments were used to characterize spontaneous and intermittent partial unwrapping dynamics of nucleosome. DNA unwrapping occurs from outer stretches and the rate constants for unwrapping depends on the distance inside the nucleosome [17]. The dissociation constant for unwrapping also depends on salt concentration; as expected, higher salt concentration enhances unwrapping. Due to the spontaneous site-exposure, DNA-binding proteins can access to nucleosomal DNA. Mechanical unwrapping experiments clarified site-dependent interaction between DNA and histone cores [20]. In addition to the central dyad region where the strongest interaction has been identified, the off-dyad region which is 1/2 turn from the dyad offers another strong interaction sites [20,25].

These experiments give, albeit un-ambiguous, structurally limited information for, at most, a few FRET pair distances. For example, how unwrapping dynamics is correlated with flexible histone tail dynamics is not directly observed. As a complementary approach, higher resolution structural dynamics analysis is desired. In this sense, molecular dynamics (MD) simulations are potentially powerful because they provide full of time-dependent structural information. Yet, conventional atomistic MD simulations reach, usually, microsecond time scales as of today, which is shorter than typical time scale of intermittent DNA unwrapping from nucleosomes. To speed up MD simulations drastically, one way is to coarse grain the molecule model. These coarse-grained (CG) MD simulations are getting more and more popular for large-scale biomolecular simulations [26,27].

For single nucleosome and nucleosome-arrays, various levels of CG models have been developed and used [28–37]. For the latter, mesoscopic modeling is particularly successful where a nucleosome or a histone octamer is treated as rigid object and linker DNA is represented by continuous string or chain of beads [28,30,33]. At single-nucleosome level, unwrapping of ends of nucleosomal DNA was investigated with a higher resolution CG model [35]. Their CG model was primarily developed to approximate near-native conformations so that larger-scale unwrapping of DNA was not treated. Related to this point, this CG model does not directly treat electrostatic interactions and thus salt-dependence of unwrapping was not investigated.

To address partial unwrapping of single nucleosome from small to large scales and in comparison with single molecule experiments, we need CG models that directly capture mechanical property of histones and DNA as well as electrostatic interactions with a higher resolution than the mesoscopic level. DNA models are desired to represent major and minor grooves, while protein models need to represent residues that are inserted into these grooves. Histone tails play crucial roles and thus need to be modeled as flexible and charged polymers. Thus, to address long-time partial unwrapping dynamics of single nucleosomes, in this paper we put forward CG MD simulations in which each amino acid in proteins is represented by one CG particle and one nucleotide in DNA is represented by three CG particles. Specifically, proteins, i.e., histone octamer, are modeled by a structure-based Go-model [38,39] and dsDNA is modeled by 3SPN.1 of de Pablo group [40,41]. The structure-based Go model is known to well approximate near-native fluctuations as well as global folding [42]. Interactions between histone octamer and dsDNA are approximated by electrostatic interactions and a structure-based contact potential. The explicit treatment of electrostatics enables us to address salt-concentration dependent unwrapping dynamics.

In this paper, brief description of computational methods is followed by simulation results of spontaneous fluctuation dynamics of single nucleosome. We observed multiple and salt-dependent intermediate states of unwrapping. Then, we investigate histone tail conformations in these dynamics. Moreover, we mimic histone tail acetylation by deleting charges in tails and investigate their effects on partial unwrapping. Finally, we investigate mechanical unwrapping of single nucleosome.

## Methods

### Coarse-grained protein and DNA models

We employed coarse-grained (CG) models for proteins and DNA. For the protein, we used a simple CG Go model [38,39] in which one amino acid is represented by one CG particle located at Cα position. For DNA we took a CG DNA model 3SPN.1 developed in de Pablo's group [40,41], where each nucleotide is represented by three CG particles, base, sugar, and phosphate.

The total potential energy function is divided into that for proteins, *V*_*pro*_, that for DNAs, *V*_*dna*_, and that for the interactions between proteins and DNAs, *V*_*pro-dna*_,

$$V_{total}=V_{pro}+V_{dna}+V_{pro-dna}$$

The energy function for proteins is that of Clementi et al [38],

$$\begin{array}{lll} V_{pro} & = & \sum_{i}k_{bd}^{pro}{\left(r_{i,i+1}-r_{i,i+1}^{0}\right)}^{2}+\sum_{i}k_{ba}^{pro}{\left(\theta_{i}-\theta_{i}^{0}\right)}^{2}\\ & + & \sum_{i}\left\{k_{dih1}^{pro}\left[1-\text{cos}\left(\varphi_{i}-\varphi_{i}^{0}\right)\right]+k_{dih3}^{pro}\left[1-\text{cos}3\left(\varphi_{i}-\varphi_{i}^{0}\right)\right]\right\}\\ & + & \sum_{i<j-3}^{nat\;contact}\varepsilon_{go}^{pro}\left[5{\left(\frac{r_{ij}^{0}}{r_{ij}}\right)}^{12}-6{\left(\frac{r_{ij}^{0}}{r_{ij}}\right)}^{10}\right]+\sum_{i<j-3}^{non-native}\varepsilon_{ev}^{pro}{\left(\frac{\sigma^{pro}}{r_{ij}}\right)}^{12} \end{array}$$

where the first, second, and third terms represent restraint potentials for virtual bond lengths, virtual bond angles, and virtual dihedral angles, respectively. The fourth term is the non-local contact potential that stabilizes amino acids pairs that are in proximity at the native (reference) structure. The last term represents a generic excluded volume effect. *r*_*i*,*i+1*_ stands for the distance of a virtual bond between i-th and i+1-th amino acids, *θ*_*i*_ is the i-th virtual angle made by two consecutive virtual bonds, *ϕ*_*i*_ is the i-th dihedral angle defined by three consecutive virtual bonds. *r*_*ij*_ is the distance between i-th and j-th amino acids. Those with superscripts 0 are parameters that take the values of the corresponding variables at the native (reference) structure. The coefficients *k*'s and *ε*'s are parameters that modulate relative balance among the terms. We used a default set of these parameter values in CafeMol [42].

The energy function for DNA is 3SPN.1 developed in de Pablo's group and can be written as

$$\begin{array}{l} V_{dna}=\sum_{i}\left[k_{bd1}^{dna}{\left(r_{i,i+1}-r_{i,i+1}^{0}\right)}^{2}+k_{bd2}^{dna}{\left(r_{i,i+1}-r_{i,i+1}^{0}\right)}^{4}\right]+\sum_{i}\frac{k_{ba}^{dna}}{2}{\left(\theta_{i}-\theta_{i}^{0}\right)}^{2}\\ \;\;\;\;\;\;\;+\sum_{i}k_{\varphi }^{dna}\left[1-\cos\left(\varphi_{i}-\varphi_{i}^{0}\right)\right]+\sum_{i<j}^{stack}4\varepsilon_{st}^{dna}\left[{\left(\frac{r_{ij}^{0}}{r_{ij}}\right)}^{12}-{\left(\frac{r_{ij}^{0}}{r_{ij}}\right)}^{6}\right]\\ \;\;\;\;\;\;\;+\sum_{i<j}^{base}\varepsilon_{bp_{i}}^{dna}\left[5{\left(\frac{\sigma^{bp_{i}}}{r_{ij}}\right)}^{12}-6{\left(\frac{\sigma^{bp_{i}}}{r_{ij}}\right)}^{10}\right]\\ \;\;\;\;\;\;\;+\sum_{i<j}^{non-native}\{\begin{array}{c} 4\varepsilon_{ev}^{dna}\left[{\left(\frac{\sigma^{dna}}{r_{ij}}\right)}^{12}-{\left(\frac{\sigma^{dna}}{r_{ij}}\right)}^{6}+\frac{1}{4}\right],\;if\;r_{ij}<2^{\frac{1}{6}\varepsilon^{dna}}\\ 0,\;otherwise \end{array}\\ \;\;\;\;\;\;\;+\sum_{i<j}^{elec}\frac{q_{i}q_{j}e^{-\frac{r_{ij}}{\lambda_{D}}}}{4\pi \varepsilon_{0}\varepsilon r_{ij}}+\sum_{i<j}^{solv}\varepsilon_{s}\left\{{\left[1-e^{-\alpha \left(r_{ij}-r_{s}\right)}\right]}^{2}-1\right\} \end{array}$$

Here, the first, second, and third terms are restraint potentials for virtual bond lengths, virtual bond angles, and virtual dihedral angles. The fourth and fifth terms are non-local contact potentials, in which the fourth one is for the stacking energy and the fifth one represents base-pairing. The sixth term is for excluded volume effect. The seventh term is a regular Coulomb energy with the Debye-Huckel screening. In the Debye-Huckel formula, *λ*_*D*_ depends on ionic strength, and thus salt concentration of the solution. The last term represents empirical solvation energy to facilitate base-pairing. Parameters with superscript 0 take values of the corresponding variables at B-type dsDNA. The rest of parameters were tuned to approximate general properties of dsDNA. We used the default parameter values of 3SPN.1, except for the dielectric constant of water *ε* that was simplified as the constant value 78. See the original article for other details [41]. We note that the Debye-Huckel model is a computationally efficient, but crude approximation for such a highly charged molecule as DNA and the explicit treatment of counter ions provides higher resolution and probably more accurate estimates of electrostatics in DNA [36,43].

Modeling interactions between the histone octamer and dsDNA is not straightforward. If the interaction was very specific and many of side-chains of histones perfectly fit with DNA, using the structure-based potential, i.e., Go-potential would be reasonable. If, on the other hand, the interaction was purely non-specific, the generic electrostatic interaction alone would be reasonable, as in our earlier work of p53 [44]. We note that the single-molecule experiments often use the so-called Widom 601 DNA sequence, a selected high-affinity nucleosome positioning sequence, of which specificity is clearly non-negligible, but incomplete as well. The current DNA model does not account for detailed sequence dependent property of DNA. To this end, we decided to include a weakened Go-potential, in which the scaling parameter is tuned so that the resulting dynamics matches some of experiments.

The interactions between proteins and DNA include the electrostatic interaction in the same way as that in DNA, the general excluded volume interactions in the same form as in the protein model, and structure-based pairwise contact potential that stabilize protein-DNA complex in a reference structure, which in the current case, a crystal structure of nucleosome,

$$\begin{array}{lll} V_{pro-dna} & = & \sum_{i<j}^{nat\;contact}\varepsilon_{go}^{pro-dna}\left[5{\left(\frac{r_{ij}^{0}}{r_{ij}}\right)}^{12}-6{\left(\frac{r_{ij}^{0}}{r_{ij}}\right)}^{10}\right]\\ & + & \sum_{i<j-3}^{non-native}\varepsilon_{ev}^{pro-dna}{\left(\frac{\sigma^{pro-dna}}{r_{ij}}\right)}^{12}+\sum_{i<j}^{elec}\frac{q_{i}q_{j}e^{-\frac{r_{ij}}{\lambda_{D}}}}{4\pi \varepsilon_{0}\varepsilon r_{ij}} \end{array}$$

The first term is the structure-based contact term, in which the parameter $\varepsilon_{go}^{pro-dna}$ controls specific attraction between histone proteins and DNA, while the last term provides sequence-non-specific attraction between positively charged histone amino acids and DNA. In the structure-based contact term, we used sugar and base sites in DNA, but not including phosphate sites because the phosphate is a charged group and is primarily represented by its charge. For charges *q*_*i*_, we assigned the standard ionization states; namely, all phosphates group in DNA, all the Glu, and Asp residues have -1, and Lys, Arg, and His possess +1 charges. We tested the case that all His charges equal to zero finding that the difference in DNA unwrapping between protonated (charged) and deprotonated (uncharged) His is rather minor (S3 Fig). The difference appears only in free energy depths of high energy meta-stable states.

For a quick test of the model, we calculated the root mean square fluctuations (RMSFs) and compared them with the experimental B-factors (S1 Fig). For histones and DNAs, the RMSFs as a function of residues reproduced major features of the experimental B-factors. Quantitatively, the overall correlation coefficient was 0.80.

In summary, our protein and DNA models are identical to those that have been used in literature, while the specific interaction between the histone octamer and DNA contains, in addition to standard terms, one new parameter $\varepsilon_{go}^{pro-dna}$ of which value needs to be calibrated.

### Nucleosome

**Fig 1:**
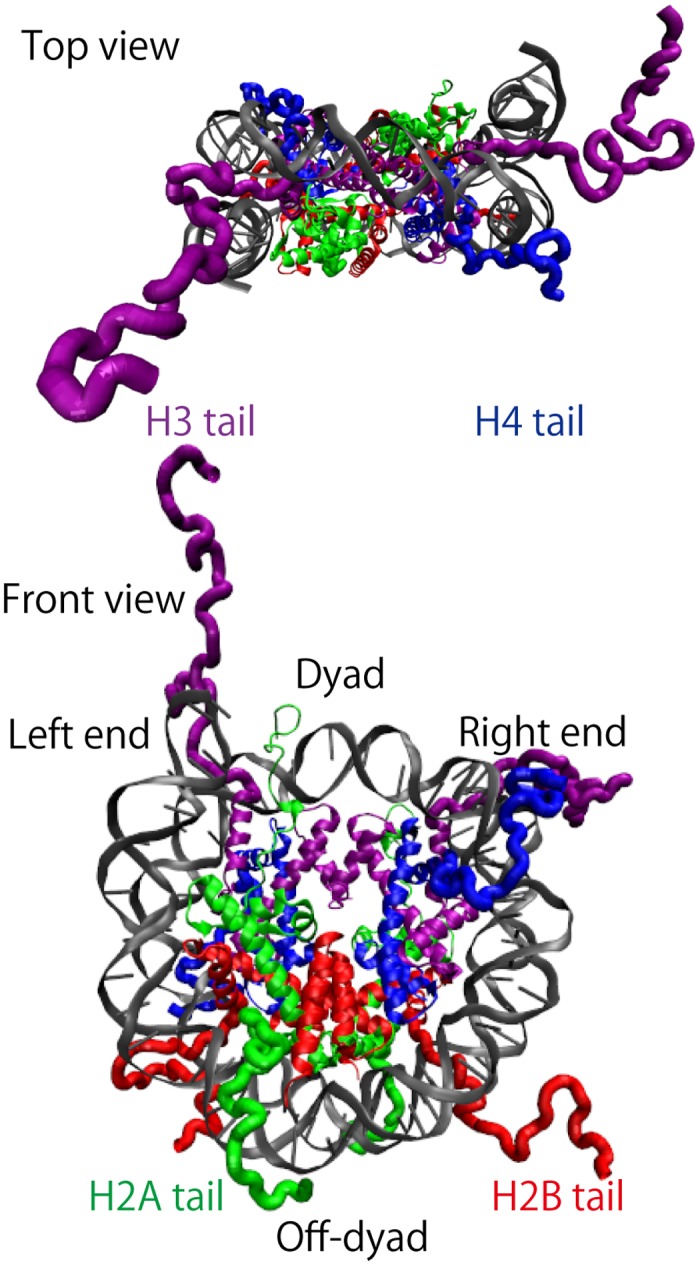
X-ray crystal structure of nucleosome (pdb code 1KX5.pdb). Top view (top) and front view (bottom) are given. Histone N-terminal tails are drawn by a tube model. H3 is in purple, H4 in blue, H2A in green, H2B in red, and dsDNA in grey. It contains 147-bp of dsDNA, of which the left (right) end is located at the top left (right) in the front view. Histone-DNA interactions are known to be strong at dyad and off-dyad regions. Note that H3 tail (1–44) and H2B tail (4–36) pass through clefts between two dsDNAs, while H4 tail (1–30) and H2A tail (1–26) extend to the side of nucleosome and pass along a single dsDNA. In H2B tail, we did not include the first three amino acids, PEP, in all the simulations, because they are removed in the crystal structure.

The nucleosome we simulated is the same molecular complex as the X-ray crystal structure with the pdb code 1KX5 [2,4] (Fig 1). The complex contains dsDNA of 147-bp and a histone octamer. The DNA sequence is palindromic taken from one-half of a human a-satellite sequence repeat. The histone octamer is from *Xenopus laevis*. Histone tails are explicitly included except the first three residues (PEP) of H2B that are missing in the pdb data. The crystal structure is pseudo-symmetric for 180 degree rotation around an axis that goes through the dyad, the central part of dsDNA. The same pdb structure was used as the reference structure in the structure-based model.

### Molecular dynamics (MD) simulations

The equation of motion that drives the system is the standard Langevin equation,

$$m_{i}\frac{d^{2}r_{i}}{dt^{2}}=-\frac{\partial V_{total}}{\partial r_{i}}+m_{i}\gamma_{i}\frac{dr_{i}}{dt}+m_{i}\xi_{i}$$

in which the random noise ξ_i_ is the Gaussian white noise with the mean and variance,

$$\langle \xi_{i}\left(t\right)\rangle =0,\;\langle \xi_{i}\left(t\right)\xi_{j}\left(t\prime \right)\rangle =\frac{2\gamma_{i}k_{B}T}{m_{i}}\delta \left(t-t\prime \right)\delta_{i,j}$$

respectively. Here, *m*_*i*_ is the mass, for which we used CafeMol default value. *k*_*B*_ is the Boltzmann constant, and *T* is the temperature set as 300K. *γ*_*i*_ is the friction coefficient and we used very low value (0.02 in CafeMol unit) to speed up the dynamics. The unit of time is denoted as t_0_. An apparent mapping leads to *t*_*0*_ ≈ 0.2 ps although the dynamics realized is known to be accelerated by many factors associated with coarse graining; the absence of side chain atoms, the absence of explicit water molecules, ignorance of hydrodynamic effects, the low friction coefficient, and so on. A time step of 0.1*t*_*0*_ was used for time integration. Each MD simulation contains 10^8^ time steps up to 10^7^ *t*_*0*_ time, unless otherwise denoted. In each case, we repeated MD 20 times with different random noises to obtain the structural ensembles. To define which part of dsDNA is unwrapped from the histone octamer, we calculated the deviation *d*_*DX*_ of every base pairs in dsDNA from those in the reference X-ray structure in each snapshot. We define the n-bp unwrapped state by that the deviations *d*_*DX*_ of 1 to n-th bp from each end of dsDNA are larger than 10 Å and that of n+1-th bp is smaller than 10 Å. We did not impose a box boundary so that, once the histone octamer and DNA dissociates, they do not re-assemble in general. In the forced unwrapping simulation of DNA from nucleosome, in each of two ends of dsDNA, we introduced a virtual particle that is linked to terminal particles by harmonic bonds. Here, terminal particles in each end of dsDNA include 5’ end of one strand and 3’ end of the other strand. We pulled the virtual particles with a constant velocity in opposite directions. The pulling velocity was 10^−4^ Å*t*_*0*_^*-1*^. In this forced unwrapping simulations, we needed slightly different parameters in MD: time step was reduced to 0.05*t*_*0*_ due to a large pulling force. Each MD simulation contains 10^8^ time steps up to 0.5·10^7^ *t*_*0*_ time. In each salt concentration case, we repeated MD runs 20 times with different random noises. The force was measured from the lengths of the harmonic bonds attached to the virtual particles. We note that, unavoidably, the pulling speed is orders of magnitude faster than that in referred experiments.

All the MD simulations were performed by CafeMol [42].

## Results/Discussions

### Partial unwrapping

The degree of DNA unwrapping is expected to depend on the strength of interaction between the histone octamer and DNA, in which, as described in Methods, an appropriate value of the interaction parameter $\varepsilon_{go}^{pro-dna}$ is unknown beforehand. Thus, we conducted preliminary simulations of thermal fluctuations of single nucleosome with various values of $\varepsilon_{go}^{pro-dna}$ in the range $0\leq \varepsilon_{go}^{pro-dna}\leq 1.0\varepsilon_{go}^{pro}$. Here, we mostly present the results with $\varepsilon_{go}^{pro-dna}=0.8\varepsilon_{go}^{pro}$, which turned out to be a representative value comparing with experimental data [17]. In [17], Widom and his collaborators performed the FRET experiments to monitor spontaneous DNA unwrapping and subsequent rewrapping. The Cy3 donor was introduced in one of several DNA positions, while the Cy5 acceptor was connected to histones so that its distance from the donor was small enough in the wrapped state. Measuring the FRET efficiency for a range of salt concentration, they obtained both kinetic and thermodynamic parameters for partial unwrapping of different levels. In equilibrium, the end of DNA, on average, started unwrapping at the salt concentration ~250 mM, while global unwrapping was observed at and above ~750 mM. As described below, we confirmed that these overall feature can be reproduced with $\varepsilon_{go}^{pro-dna}=0.8\varepsilon_{go}^{pro}$. (Later, to see the dependence of the parameter $\varepsilon_{go}^{pro-dna}$, we show some results of $\varepsilon_{go}^{pro-dna}=0.5\varepsilon_{go}^{pro}$, and 1.0 $\varepsilon_{go}^{pro}$).

**Fig 2:**
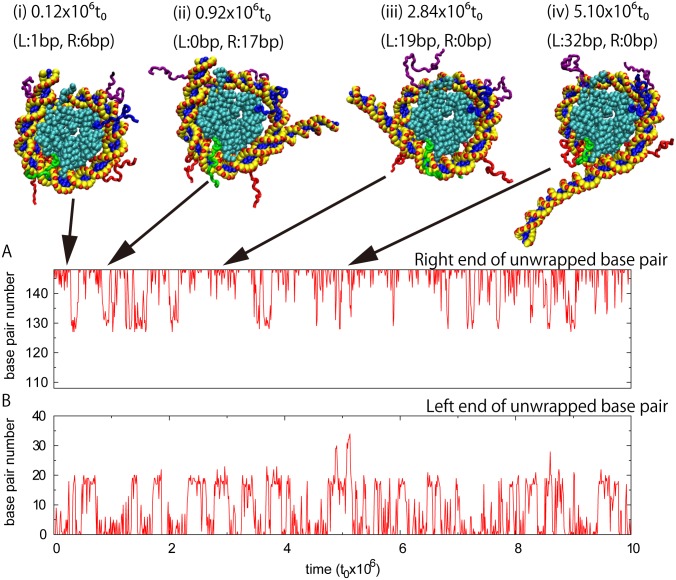
A typical time course of coarse-grained molecular dynamics simulations of a nucleosome. Simulations were performed at NaCl concentration 300 mM with the default histone-DNA interaction strength $\varepsilon_{go}^{pro-dna}=0.8\varepsilon_{go}^{pro}$. The vertical axis is numbered from the left end of dsDNA. DNA transiently and reversely showed partial unwrapping. In each snapshot, a consecutive segment of DNA is wrapped to the histone octamer core. Panels A and B plot time courses of unwrapped DNA segments in the right and the left ends, respectively. Four representative snapshot structures (i)-(iv) are drawn at the top with the time and the assigned left and right ends of the unwrapped region in parentheses.

MD simulations of nucleosome at NaCl 300 mM showed repeated partial unwrapping in both the left and right ends of dsDNA (Fig 2). Note that the definition of "left" and "right" is arbitrary and that the bp number runs from 1 in the left end to 147 in the right end. We use them merely to distinguish two ends of dsDNA throughout this paper. In each of snapshots, a central consecutive segment of dsDNA was bound to histone octamer, while the left and/or right ends of dsDNA may be transiently unwrapped. We thus can define, in each snapshot, the first bp of the right unwrapped dsDNA segment and the last bp of the left unwrapped dsDNA segment, of which base pair numbers are illustrated in Fig 2A and 2B, respectively, for a typical trajectory. In preliminary investigation, we explored some other measures, such as distances between chromophore attaching sites in FRET experiments, and angles between two ends of DNA, to quantify the degree of partial unwrapping of DNA. These parameters showed a clear peak when DNA is fully wrapped and a broad distribution when DNA is partially unwrapped due to fluctuation of unwrapped DNA segments. Seeking a better measure that is not subject to such fluctuations of unwrapped DNA and thus is more sensitive, we reached the order parameter defined above.

Clearly, in Fig 2, partial unwrapping occurred repeatedly and transiently. Patterns of unwrapping in the left and right ends were not significantly different, probably due to its pseudo-symmetry in the crystal structure. The figure also suggests that partial unwrapping in each end takes some distinct states. One obvious state assigned is the zero-bp unwrapped state. Among snapshot structures depicted in Fig 2, those in (i) and (ii) have their left ends in the zero-bp unwrapped state. Another clear state observed has about 20-bp unwrapped, which is illustrated in the snapshot (ii) for its right end and the left end of the snapshot (iii). Rather rarely, we observed the third state where about 30-bp are unwrapped as in the snapshot (iv) for its left end. Moreover, we notice that there seem a marginal state with about 10-bp unwrapped.

Partial unwrapping in the left and right ends seems to occur independently. A statistical analysis failed to find any noticeable correlation between unwrapping of two ends (S2 Fig). The degree of correlation, if any, is expected to depend on the salt concentration of the solution because, with a low salt concentration, we expect to have relatively long-range electrostatic interactions. Yet, even at the lowest salt concentration studied here (see below), we did not find any crucial correlation of partial unwrapping in two ends (S2 Fig).

**Fig 3:**
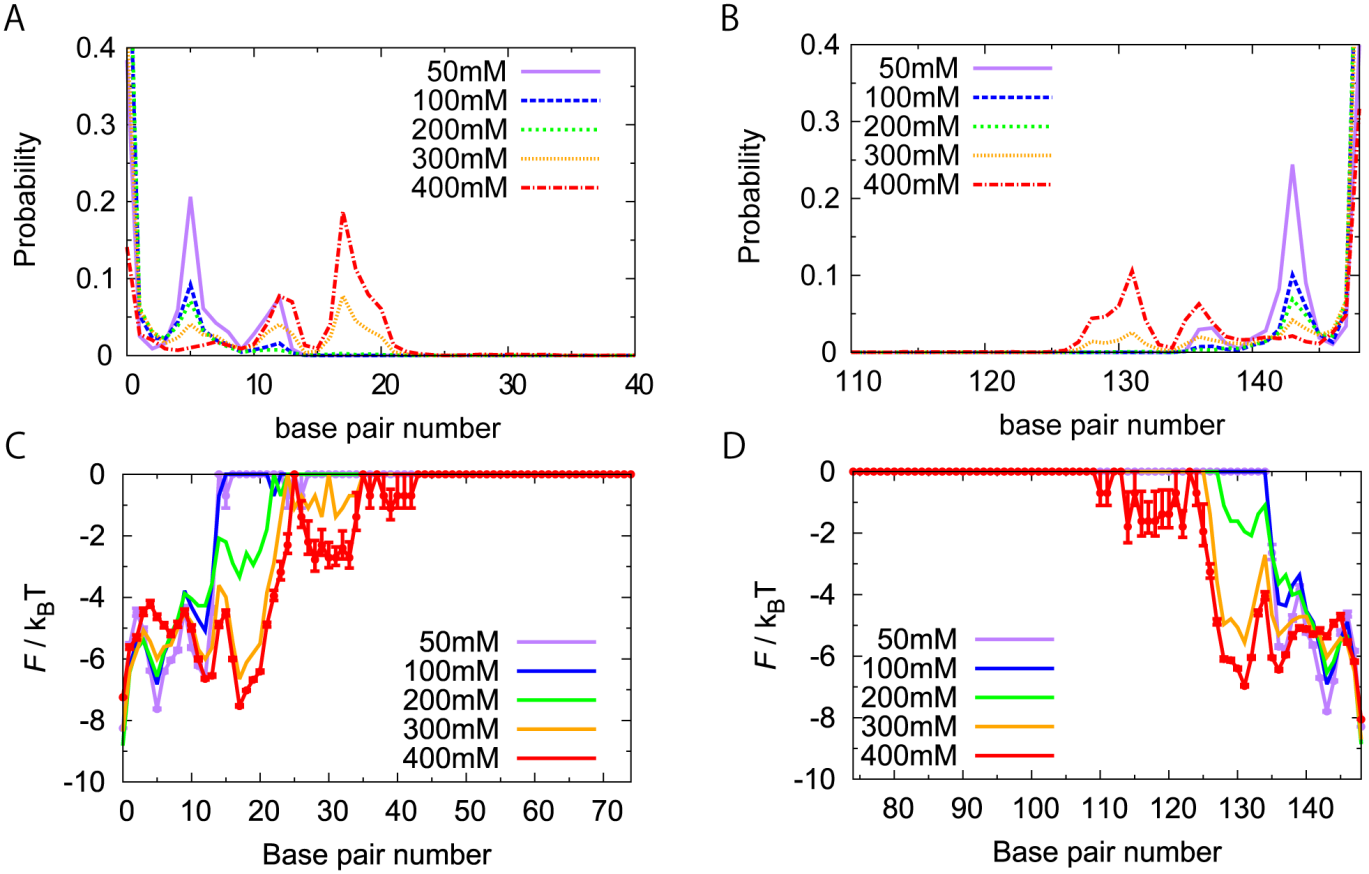
Statistics and stability of partially unwrapped states of nucleosome. The horizontal axis is numbered from the left end of dsDNA. (A,B) The probabilities of the left (A) and right (B) ends of the unwrapped DNA segment. (C,D) The corresponding "free energy profile" for the left (C) and right (D) ends of unwrapped regions. For clarity, we put error bars only for the results of 50 mM and 400 mM to exemplify the statistical errors. The free energy equal to zero means that we did not sample that region (For this region, the error bar was omitted). Results are drawn for five different salt concentrations, NaCl 50 mM (purple), 100 mM (blue), 200 mM (green), 300 mM (orange), and 400 mM (red). Note that, above 500 mM, global unwrapping (dissociation) happened which hampered to obtain the converged histograms and free energy profiles.

To quantify partially unwrapped states, we obtained probabilities *P(bp)* of the left and right ends of the unwrapped DNA segment from 20 trajectories, which are shown in Fig 3A (the left end) and Fig 3B (the right end). Equivalently, we also plotted free energy profiles, which are defined as −ln(*sample_number* × *p*(*bp*) + 1) in Fig 3C (the left end) and Fig 3D (the right end). At 300 mM NaCl condition (orange), we see two dominant minima in free energy profile, one at zero-bp and the other at 17-bp unwrapped states. We also find shallower minima at 5-bp and 12-bp unwrapped states, as well as a marginal state at ~ 30-bp unwrapped state. We note that, from the crystal structure, one would expect to have 5-bp periodicity. The histogram shows rather close, albeit not identical, 5-bp periodicity. We note that high free energy part of the profile that was not sampled in the current simulations can be better estimated by use of some enhanced sampling techniques.

In the same way as above, we obtained free energy profiles of the left and right ends of unwrapped bp for various different salt concentrations between 50 mM and 400 mM NaCl (Fig 3C and 3D). At salt concentrations equal to and higher than 500 mM, dsDNA progressively unwrapped and finally dissociated from the histone octamer. We note that the simulation did not account for the re-assembly process. At these conditions, we cannot well-define the free energy profiles and thus we did not plot them. Between 50 mM and 400 mM, as expected, at a higher salt concentration dsDNA tends to be unwrapped more. At 50 mM and 100 mM, we see three distinct states; zero-bp unwrapped as the dominant state, 5-bp, and 12-bp unwrapped states as minor states. At 200 mM, on top of the above three states, we also see the fourth state with ~17-bp unwrapped. At 300 mM and 400 mM, the state with 17-bp unwrapped became the major state, still retaining the above mentioned unwrapped states as metastable states. Thus, as the salt concentration changes, the positions of local minima are mostly kept fixed, while their relative stabilities vary.

DNA unwrapping involves large energy-entropy compensation, somewhat similar to that in protein folding [45]. In comparison with protein folding processes, DNA unwrapping seems less cooperative producing a series of partially unwrapped sub-states. This could be attributed to less flexibility of unwrapped dsDNA than unfolded proteins.

### Weaker and stronger specific interactions between DNA and histone octamer

Next, we investigate how unwrapping is affected by the change in the interaction strength between DNA and histone octamer. All the above simulations used the coefficient $\varepsilon_{go}^{pro-dna}=0.8\varepsilon_{go}^{pro}$. Here, we tested a weaker interaction $\varepsilon_{go}^{pro-dna}=0.5\varepsilon_{go}^{pro}$ and a stronger interaction $\varepsilon_{go}^{pro-dna}=1.0\varepsilon_{go}^{pro}$.

**Fig 4:**
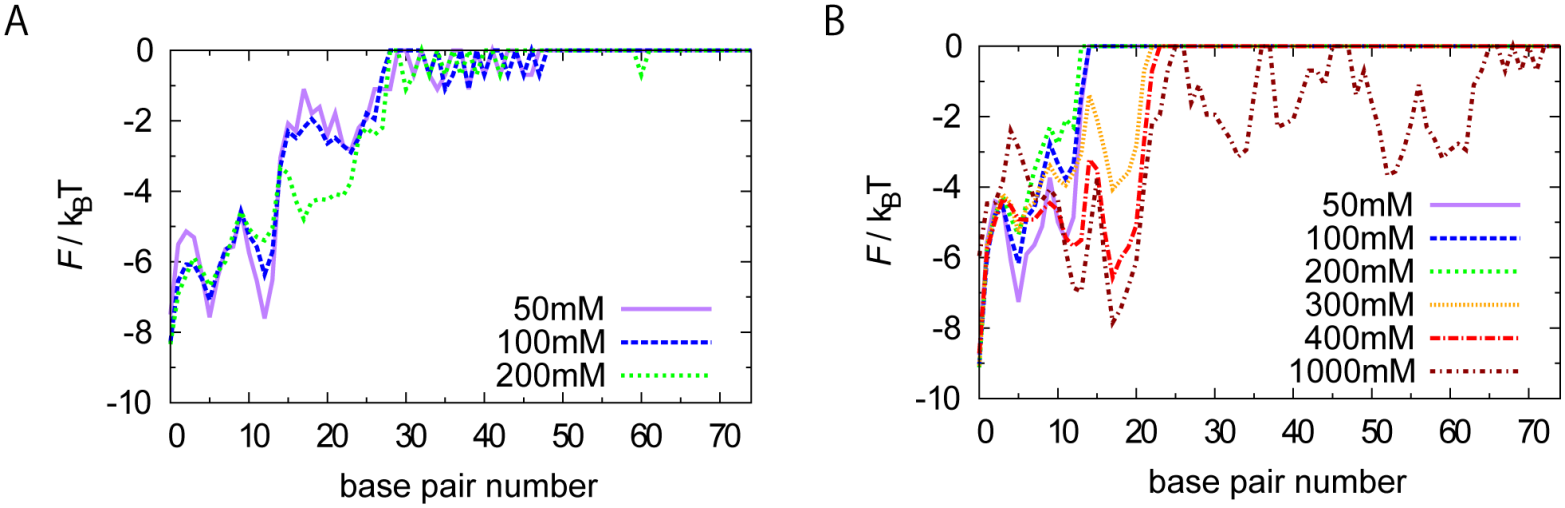
Sensitivity test of the structure-based interaction parameter. Free energy profiles for the left end of unwrapped DNA are plotted for a weakened interaction, $\varepsilon_{go}^{pro-dna}=0.5\varepsilon_{go}^{pro}$ (A) and a strengthened interaction $\varepsilon_{go}^{pro-dna}=1.0\varepsilon_{go}^{pro}$ (B). The default parameter used for all the other simulations is $0.8\varepsilon_{go}^{pro}$. With the parameter $\varepsilon_{go}^{pro-dna}=0.5\varepsilon_{go}^{pro}$, DNA is completely unwrapped and dissociated from histone octamer at and above 300 mM NaCl. With the parameter $\varepsilon_{go}^{pro-dna}=1.0\varepsilon_{go}^{pro}$, DNA did not dissociate from histone octamer within the computing time even at a sufficiently high salt concentration such as 1 M.

Fig 4A shows the free energy profile of the left end of wrapped DNA for the weakened case, $\varepsilon_{go}^{pro-dna}=0.5\varepsilon_{go}^{pro}$ for salt concentration NaCl 50 mM, 100 mM, and 200 mM. While the overall shapes of the free energy profiles look similar to the default one, the zero-bp unwrapping state was less stable while 5-bp, 12-bp, and 17-bp unwrapped states were more stable. Furthermore, at salt concentration higher than 200 mM, after partial unwrapping, dsDNA was completely dissociated from the histone octamer. These data suggest that $\varepsilon_{go}^{pro-dna}=0.5\varepsilon_{go}^{pro}$ lead to less stable nucleosome than the stability experimentally suggested for high affinity sequences, such as Widom's 601 sequence [17], where the average structure showed no partial unwrapping below 250 mM.

Next, we plotted the free energy profile for the left end of wrapped segment in the case of the stronger interaction $\varepsilon_{go}^{pro-dna}=1.0\varepsilon_{go}^{pro}$. As expected, we observed partial unwrapping less frequently than the default case (Fig 4B). Yet, at the salt concentration NaCl 400 mM, we observed partial unwrapping up to 17-bp. Even at the salt concentration of NaCl 1 M, we did not observe any complete unwrapping/ dissociation from DNA. This suggests that, with the strong interaction parameter, the structure-based Go potential alone can stabilize nucleosome no matter how the salt concentration is high within the simulation time scale. This is inconsistent with experimental results since experimentally most nucleosomes are disassembled at the salt concentration as high as 1 M [17].

These surveys of interaction parameter led us to use $\varepsilon_{go}^{pro-dna}=0.8\varepsilon_{go}^{pro}$ as the default value in the current work. We note that the affinity of nucleosomal DNA with the histone octamer has a broad range that is more than a thousand, and thus it is not very important to fine tune the interaction parameter. $\varepsilon_{go}^{pro-dna}=0.5\varepsilon_{go}^{pro}$ may correspond to a weak affinity sequence, while $\varepsilon_{go}^{pro-dna}=1.0\varepsilon_{go}^{pro}$ is for a high affinity sequence. Hereafter, we solely use the default value.

### Conformations of histone tails

**Fig 5:**
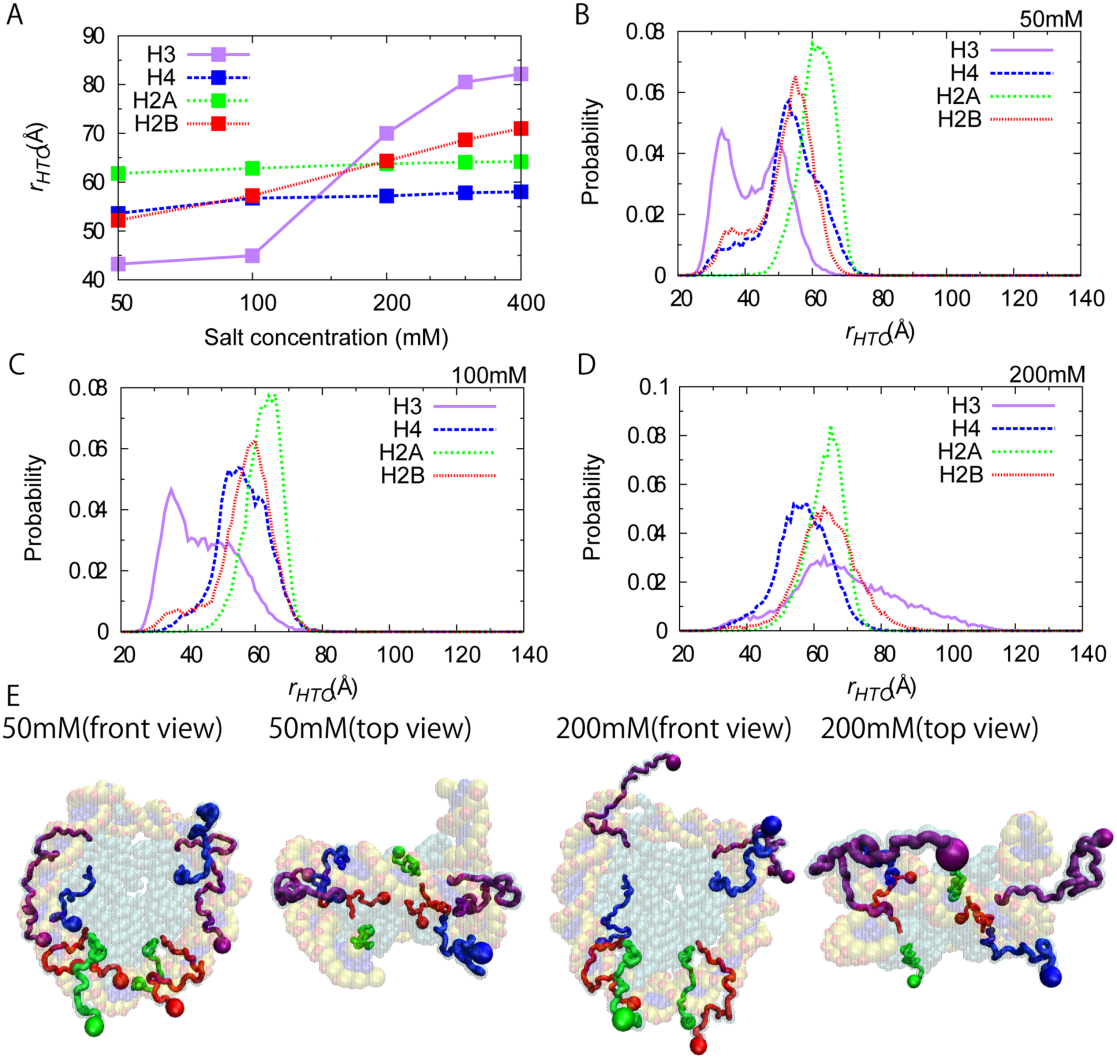
Conformation of histone tails. (A) Average distances *r*_*HTO*_ between histone N-terminus residues and the center of histone octamer core at various salt concentrations. (B,C,D) The distribution of *r*_*HTO*_ for four histone tails at NaCl 50 mM (B), at 100 mM (C), and at 200 mM (D). (E) Representative snapshots of histone tails. The terminal residues are represented by spheres. The color assignments are the same as Fig 1.

It is interesting to look into conformations of histone tails, which can be correlated with partial unwrapping of DNA. We first plotted the distances *r*_*HTO*_ of the histone tail terminal residues from the center of mass of histone octamer core (Fig 5A), averaged over trajectories. We clearly see that, for all histone tails, the tail termini become more distant from the center as the salt concentration increases. This is an expected result because highly basic histone tails are attracted to DNA, and the attraction is stronger at lower salt concentration. At a higher salt concentration, these attractions are weaker and disordered tails take entropically extended conformations. These results are in good agreement with recent simulation results [36]. In Fig 5A, we see that the salt concentration dependence of the distance is markedly stronger for H3 tails than the other three tails.

To understand conformations and interactions of histone tails further, we plotted the probability distribution of the distance *r*_*HTO*_ for some salt concentrations (Fig 5B–5D). At 50 mM, *r*_*HTO*_ for H3 tails exhibited a bimodal distribution with two peaks at ~35 Å and at ~50 Å. Even for H4 and H2B, we marginally recognize two states, a major state centered at ~60 Å and a minor one around 35 Å. On the other hand, *r*_*HTO*_ for H2A has only one peak centered at ~60 Å. Looking at snapshot structures, we identified that 35 Å from the center of histone core corresponds to the boundary between the histone octamer core and the wrapped DNA, which makes concave surface. At 50 mM, many of histone tail terminals were located at the concave surface. We illustrated one snapshot in the left cartoon of Fig 5E where the left H3 tail and the left H2B tail terminals are located in the boundary between histone octamer and DNA. On the other hand, the distance *r*_*HTO*_ = 50–60 Å corresponds to the case that histone tails are located near the outer surface of wrapped DNA, which can be seen in the left and right H3 tails and the right H4 tail in the left cartoon of Fig 5E. At higher salt concentration, the *r*_*HTO*_ distributions generally moved to the larger distance (Fig 5C and 5D). In particular, at the salt concentration 200 mM all the histone tail terminals show single peak near ~60 Å. Interestingly, the H3 tail has broader distribution than the others perhaps because of its length. In these long distances, the histone tail terminal is not in contact with histone cores or DNA. Instead, at 200 mM, histone tails fluctuate apparently randomly. We note that the use of the Go model in the local potential energy function for disordered tails might have made the tails somewhat more rigid than they are. Yet, we confirmed that, with use of the flexible loop modeling [46], the results are not significantly different (S4 Fig).

### Correlation between DNA unwrapping and histone tail dynamics

**Fig 6:**
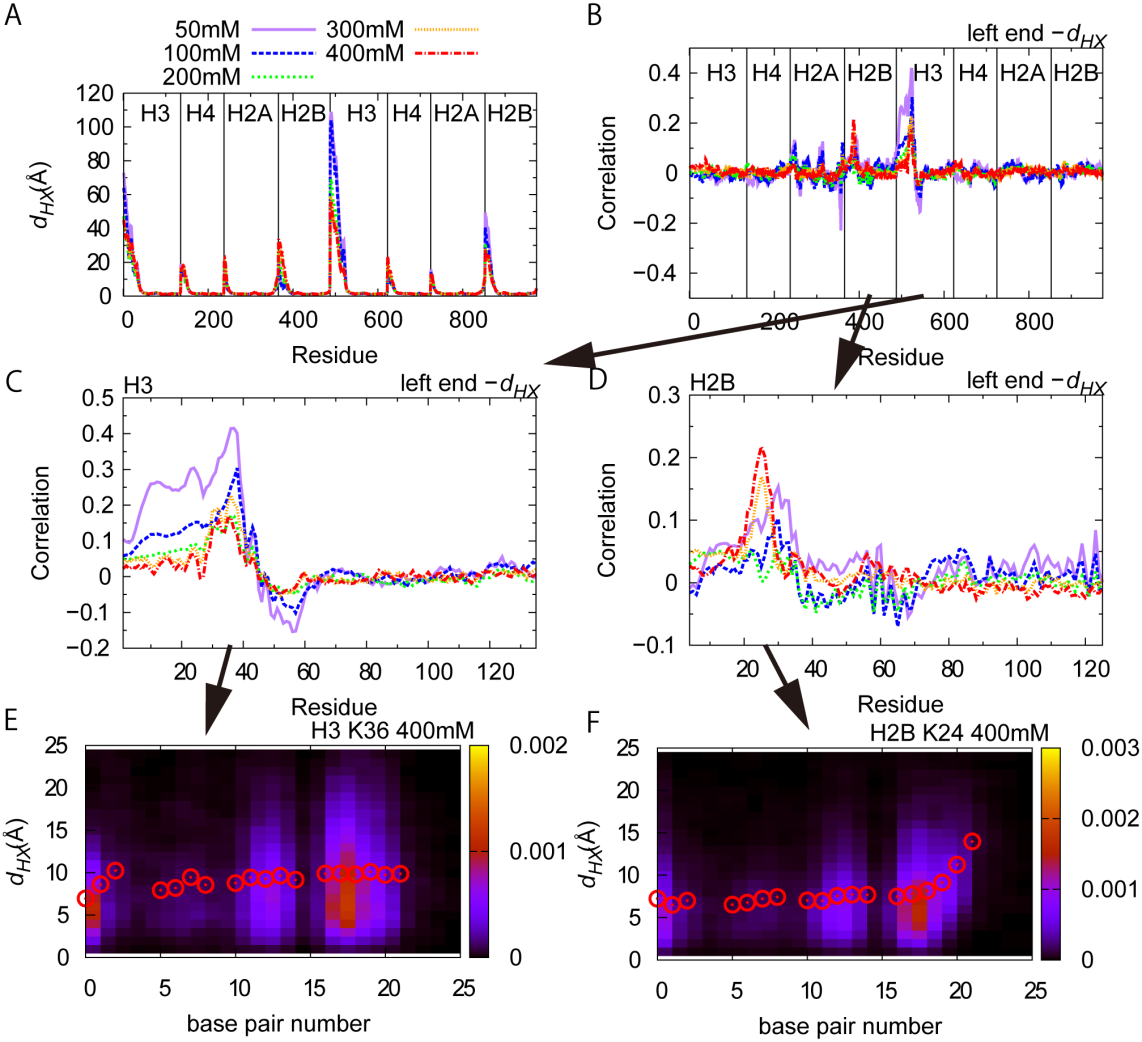
Correlation between partial unwrapping of DNA and fluctuation of histone tails. (A) Average deviations *d*_*HX*_ of individual histone residues between the simulated and the X-ray structure at various salt concentrations. (B) Correlation coefficients of *d*_*HX*_ and the left end of the unwrapped DNA segment. (CD) (B) is enlarged at the N-terminal tails of H3 (C), and H2B (D). (EF) The distribution of the left end of the unwrapped DNA segment and the displacement *d*_*HX*_ of H3K36 (E) and H2B K24 (F). Red circles show the average values of *d*_*HX*_.

Next, we investigate correlation between DNA partial unwrapping and histone protein fluctuations. First, we calculated the deviation *d*_*HX*_ of every amino acids in histone octamer from those in the reference X-ray structure in each snapshot. The average of *d*_*HX*_ over 20 trajectories is plotted in Fig 6A. As expected, relatively large deviation/fluctuation is localized to N-terminals. Then, we obtained the correlation coefficients between *d*_*HX*_ and the unwrapped DNA bp in the left end, which is shown in Fig 6B. We find that the correlation coefficient is relatively large for one of H3 and H2B tails that interact with left end of DNA. These relevant parts were magnified and re-plotted in Fig 6C and 6D. It is reasonable that H3 and H2B tails have larger effects to DNA unwrapping because these two tails have their roots at the side of nucleosome and between two turns of dsDNA. In H3, high correlation appears around 25–45 residues, which is near a root of the tail (the H3 tail corresponds to residues 1–44). In H2B, high correlation is seen around 15–35 residues, which is also close to the root of the tail (the H2B tail is assigned as 4–36 residues). These clearly indicate that the root regions of H3 and H2B tails contribute to stabilize the wrapped DNA. On the other hands, both tail terminals have less correlation to the DNA unwrapping because they are already far from the wrapped DNA and highly fluctuating.

To further clarify the coupling of root regions of H3 and H2B tails with DNA unwrapping, we plotted the distribution (and the average) of deviations *d*_*HX*_’s for H3K36 and H2B K24 as a function of unwrapped DNA bp in Fig 6E and 6F, respectively. For H3K36, the *d*_*HX*_ is distributed around 7 Å in the completely wrapped end, while the distribution becomes wider and the average increases to about 10Å when about 5-bp unwrapped. Yet, further unwrapping does not change the *d*_*HX*_ distribution significantly. Thus, the H3 tail contributes to stabilize the last ~5-bp of unwrapped DNA. On the other hand, for H2B K24, the *d*_*HX*_ distribution shifted to the large displacement when the unwrapped DNA bp reaches to ~20. Thus, H2B tail is suggested to stabilize wrapping of ~20-bp from the end, and thus is important for large-scale partial unwrapping. These results are all consistent with the X-ray crystal structure.

### Effect of histone tail modification

Here, we address effects of modification in individual histone tails on the unwrapping dynamics of nucleosome. As is well-known, N-terminal tails of histones contains quite many positively charged amino acids (K and R), which is expected to have major impact on the nucleosome stability. Acetylations of K and R reduce the positive charges in histone tails. How such reduction in tail charges affects the unwrapping dynamics are, thus, of great interest. We conducted a series of CG MD simulations where one of tails has no charge in its N-terminal tail and compare its unwrapping dynamics with that of the intact one. We note that, in each case, we deleted all the N-terminal tail charge of two molecules of each type of histone.

**Fig 7:**
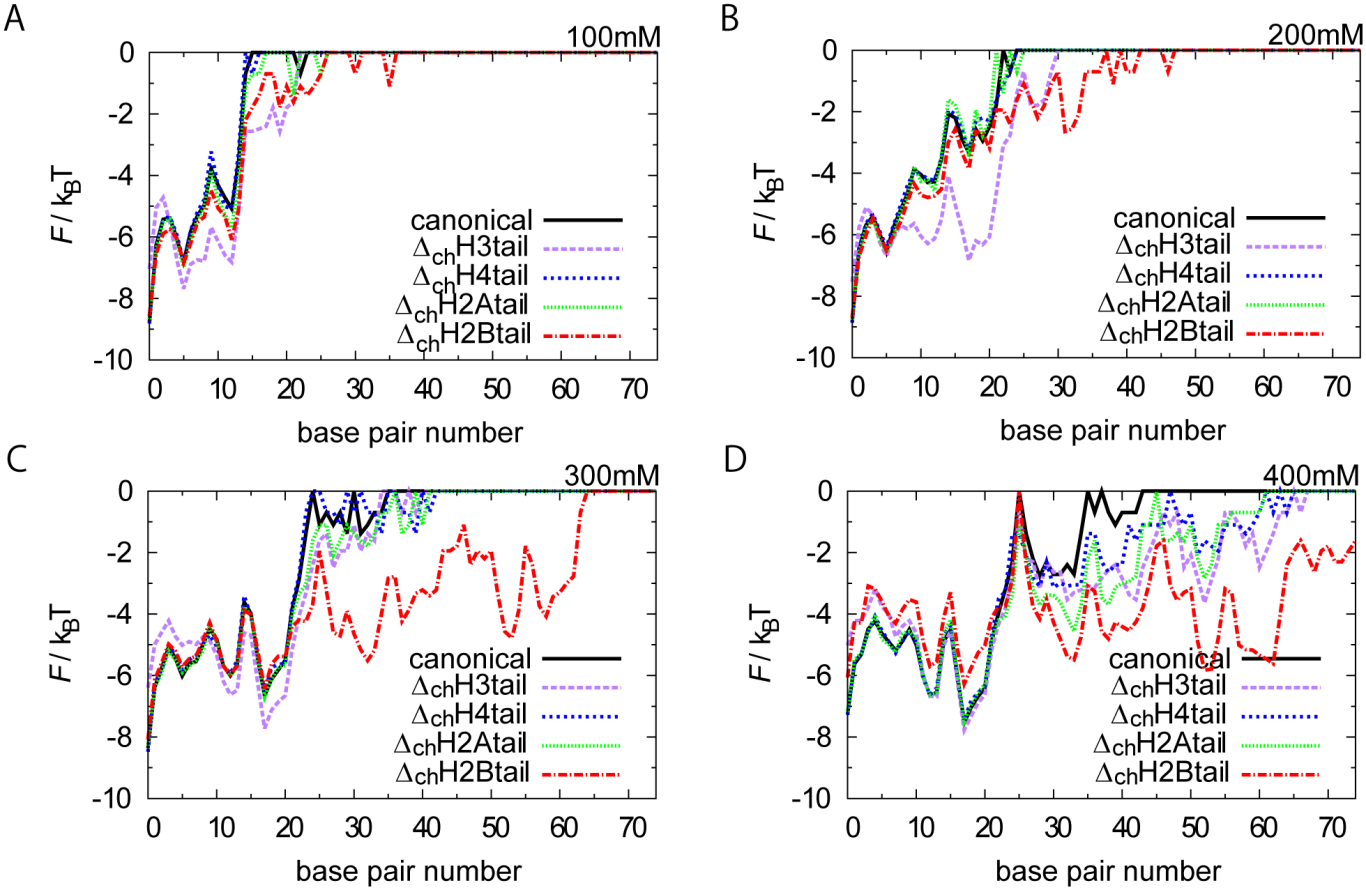
Effect of charge deletions of histone tails. Free energy profiles of the left end of unwrapped DNA for nucleosome with zero charge in one of histone N-terminal tails at NaCl 100 mM (A), at 200 mM (B), at 300 mM (C), and at 400 mM (D). Δ_ch_H3_tail_ for example stands for the nucleosome in which charge in H3 N-terminal tail is deleted in the simulations. "canonical" means the intact interaction.

Fig 7 shows the free energy profiles of individual cases, each of which has zero charge in one of the four histone tails, together with the case of the intact interaction as control (denoted as "canonical"). Overall the unwrapping was more or less enhanced by deleting charge in the tails, but the extent of enhancement depended on the cases.

The most marked effect was observed for the case that we deleted charges in the H3 tails (purple dashed curves in Fig 7). The completely wrapped state became less stable rather significantly and, instead, intermediate states with < 20-bp unwrapped became more stable than the case of the intact interaction for all the salt concentration cases tested in Fig 7. This result suggests that electrostatic interactions between H3 tail and nucleosome core contribute to stabilization of the completely wrapped state. This is reasonable because in the crystal structure (Fig 1), we see that H3 tails are located near the left and right ends of wrapped DNA.

Another tail that affects the unwrapping dynamics markedly was H2B in Fig 7 (red). In this case, the deletion of the charge did not alter stabilities of states with < 20-bp unwrapping, whereas we see dramatic increase in frequency of larger-scale unwrapping with > 20-bp unwrapped. For example, at the salt concentration 300 mM, we see partial unwrapping up to 60-bp only in the case of H2B charge deletion (Fig 7C). This suggests that the charge in H2B tails contributes to stabilize the nucleosome at around 20-bp from the ends of wrapped DNA. Again, this result is perfectly in harmony with the crystal structure where H2B tails (red in Fig 1) interact with the corresponding region of DNA.

Effects of the other two tails, H2A and H4, turned out to be weaker, as in Fig 7. These tails are extended to the side of nucleosome (Fig 1) and thus seem to little contribute to the stability of nucleosomal DNA

### Mechanical unwrapping

Finally, we performed mechanical unwrapping simulations where we attached virtual particles to both ends of DNA and pulled these virtual particles to the opposing directions with a constant velocity while monitoring the pulling force.

**Fig 8:**
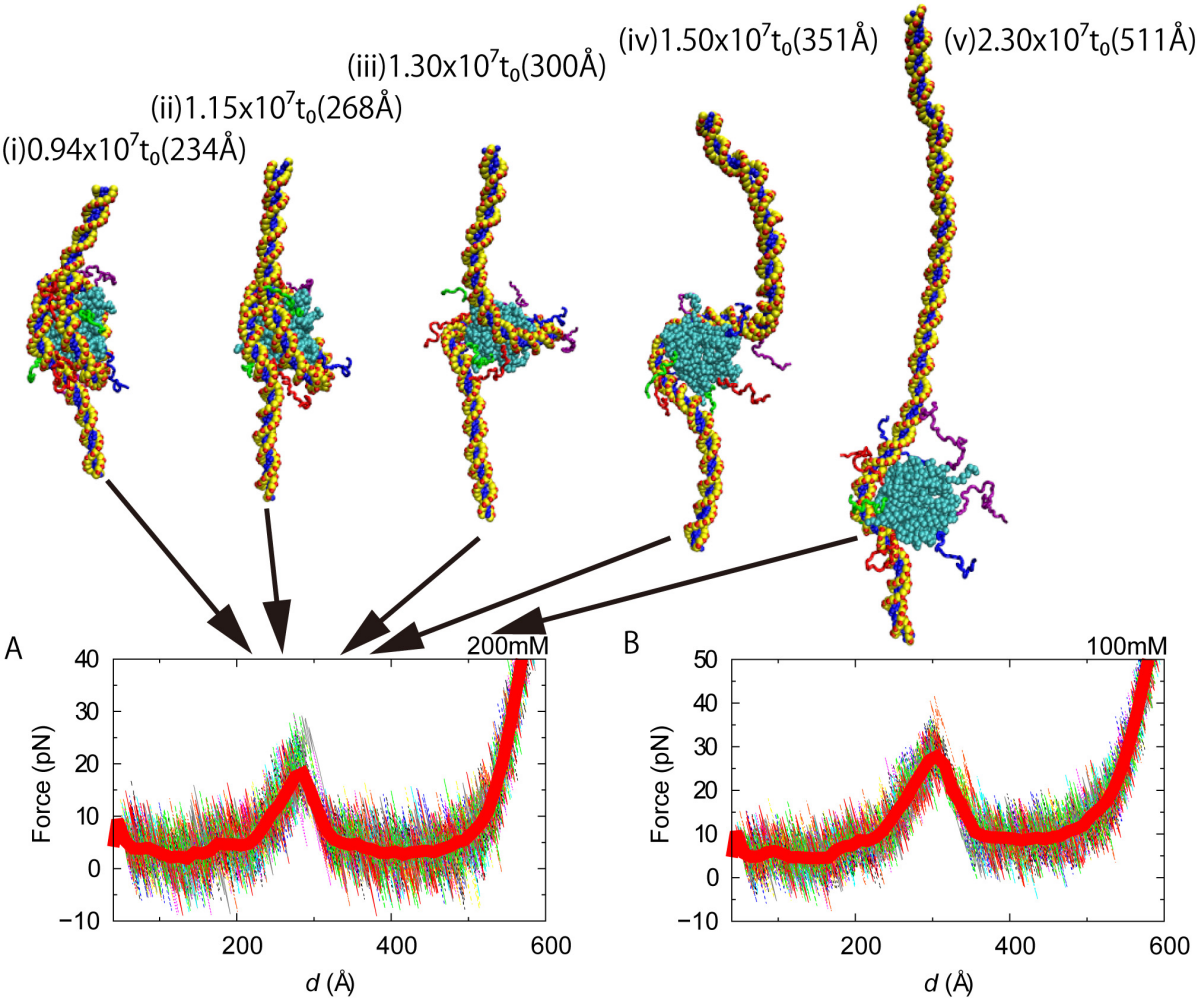
Force profiles in mechanical unwrapping of DNA from nucleosome. Results for NaCl 200 mM (A) and 100 mM (B) are plotted with some snapshot structures. 20 individual pulling trajectories are plotted by thin curves, while the average is drawn in thick red curves.

At the salt concentration 200 mM, DNA smoothly started unwrapped, and then exhibited a major barrier in the pulling force profile once 20 trajectories were averaged (Fig 8A). This result is qualitatively in good agreement with recent single molecule experiments of mechanical unwrapping [20,22,47,48]. The peak in force profile corresponds to the unbinding at the so-called off-dyad region of DNA, where histone-DNA interactions are supposed to be strong. The peak force (at *d* = 280 Å) is ~20 pN. At smaller distances, there can be one or more peaks although we cannot rule out the possibility that they are simply noise.

When we decreased the salt concentration up to 100 mM, a stronger histone-DNA interaction made the force profile slightly more structured (Fig 8B). In addition to the large peak, albeit rather faint, we see at least one small peak in the force profile when DNA started unwrapping around d = 220 Å. The peak force is ~30 pN.

Experimentally, the force associated with the major peak was 27 pN at the salt concentration 50 mM in one experiment [22]. Another measurement reported 8–9 pN for a slightly different system [47]. If we directly compare the peak forces of at the same salt concentration, the force from our simulations is somewhat larger than the experimental one. The difference may be attributed to the difference in pulling speed between the simulation and the experiment, which was previously found and argued [34]. In addition, mapping between experimental salt concentration and ionic strength in our simulations may not be quantitative.

### Conclusion

We investigated partial unwrapping dynamics of nucleosome by coarse-grained molecular dynamics simulations. Depending on the parameter for protein-DNA interaction strength, partial unwrapping dynamics is altered quite significantly. Of the three parameter values tested, we conclude that the parameter $0.8\varepsilon_{go}^{pro}$ is among the most reasonable value. With this parameter, we obtained results that are in agreement with experiments such as single molecule FRET experiment. The simulations showed spontaneous unwrapping from the outer DNA and subsequent rewrapping dynamics. We found several distinct partially unwrapped states of nucleosomes. At a low salt concentration, histone tails mostly sit in the concave cleft between the histone octamer and DNA, tightening the nucleosomal DNA. At a higher salt concentration, the tails tend to be expanded outwards, which led to higher degree of unwrapping. H3 and H2B tail dynamics are markedly correlated with partial unwrapping of DNA, and, moreover, their contributions were distinct. Acetylation in histone tails was mimicked by changing their charges, which enhanced the unwrapping, especially markedly for H3 and H2B tails.

Recently, it has been suggested that histone tail acetylation alters chromatin folding, which may be biologically more relevant effect of acetylation. The method developed here can be straightforwardly extended to poly-nucleosomes, where we can address effects of histone acetylation on the chromatin folding, which will be an interesting future direction.

Although the simulation method used here is general and can be applied to many protein-DNA complexes, the method has many limitations as well and thus there is much room for improvement. First, accurate modeling dsDNA, especially its bending flexibility, is of particular importance for accurate account of DNA unwrapping in nucleosome. Recently, de Pablo group developed a new and refined version of CG DNA model that seems to approximate sequence dependent DNA flexibility rather accurately [49]. Thus, the use of this or other refined methods may be an important extension to the current work. Second, non-specific protein-DNA interactions are dominated by electrostatic interactions, where we used a simple Debye-Huckel screening model. This model is legitimated only for dilute ionic solution of monovalent ions. However, protein-DNA interactions generally involve strong electric field that leads to locally high ion concentration. Small amount of divalent ions is known to alter chromatin folding markedly, which is clearly beyond the range of Debye-Huckel model. More refined treatment of counter ions around the molecules, such as explicit treatment of them, may be a promising direction of improvement [36].

## Supporting Information

S1 TableParameters in coarse-grained protein and DNA models.All but $\varepsilon_{go}^{pro-dna}$ values used are the standard (default) values in CafeMol.(DOCX)

S1 FigComparison between experimental and calculated fluctuations.B-factor of the X-ray crystallography (black) and that calculated from simulations (red) for (A) DNA and histones (B). For DNA, B-factor is plotted for O5’ atom in the X-ray crystallographic data and for the sugar bead in the coarse-grained model. For histones, Cα atom is used for B-factor, except for disordered regions of histone tails in the X-ray crystal structure. For simulation, salt concentration is NaCl 100 mM and $\varepsilon_{go}^{pro-dna}=0.8\varepsilon_{go}^{pro}$. The root-mean-square fluctuations of DNA are about 3–5 Å and those of histone core are about 1–2 Å; DNA beads have larger fluctuations in the simulations. (C) The correlation plot between experimental and calculated B-factors. The correlation coefficient was 0.80 for the entire system when the calculated B-factor larger than 300 were excluded. We note that the agreement for DNA is lower due to many possible reasons. The experimental B-factor is affected by crystallographic environment and global rotation, while the computation B-factor here does not take into account the crystallographic environment. Even among experimental data, B-factors in nucleosomes are largely different: Comparison of B-factors in several nucleosome crystallographic data revealed that they are little conserved overall and only the conserved feature is the periodicity of 10 bp. Importantly, the simulated B-factor reproduced this feature.(TIF)

S2 Fig Correlation between partial unwrapping of the left and right ends of DNA. Firstly, for a given snapshot, *i*-th base pair from terminus was assigned as either wrapped or unwrapped. The probability that *i*-th base pairs in both ends are in the unwrapped states were calculated (red curve designated as uu(simulations)). Under the assumption of independence of unwrapping of two ends, we estimated the expectation value that both ends are unwrapped, i.e., the product of probabilities of unwrapping of each ends (green curve designated as uu(expectation)). Results with the salt concentrations, NaCl 50 mM (A), 100 mM (B), 200 mM (C), and 400 mM (D). The correlation between partial unwrapping of the left and right end of DNA is insignificant.
(TIF)

S3 Fig Effect of histidine charge for partial unwrapping. Free energy profiles for the left end unwrapped state with histidine charge equal to +1 (blue) and zero (red) at NaCl 100 mM (A), at 200 mM (B), at 300 mM (C), and at 400 mM (D). Histidine residues in histone proteins are relatively few; histone octamer contains 18 histidine residues, whereas 106 arginine residues and 114 lysine residues.
(TIF)

S4 Fig Effect of the force field of disordered region in the histone tails. Histone tails are largely disordered; residues 1–32 of chain H3, 1–30 of H3’, 1–23 of H4, 1–15 of H4’, 1–14 and 121–128 of H2A, 1–12 and 122–128 of H2A’, 4–24 of H2B, and 4–26 of H2B’ are disordered. We tested different modeling for these disordered regions. “Go” (blue) means that we used the Go interaction based on the modeled structure in 1KX5.pdb (one that was used in the main text). “Del” (green) means that the Go interaction was deleted except for bond length term. “FLP” (orange) is the case that we deleted Go interaction and applied the statistical potential. Results with the salt concentrations, NaCl 100 mM (A), 200 mM (B), 300 mM (C), and 400 mM (D).
(TIF)

## References

[1] Andrews AJ, Luger K. Nucleosome Structure(s) and Stability: Variations on a Theme. Annu Rev Biophys. 2011;40: 99–117. 10.1146/annurev-biophys-042910-155329 21332355

[2] Luger K, Mäder AW, Richmond RK, Sargent DF, Richmond TJ. Crystal structure of the nucleosome core particle at 2.8 Å resolution. Nature. 1997;389: 251–260. 9305837 10.1038/38444

[3] Lowary PT, Widom J. New DNA sequence rules for high affinity binding to histone octamer and sequence-directed nucleosome positioning. J Mol Biol. 1998;276: 19–42. 9514715 10.1006/jmbi.1997.1494

[4] Davey CA, Sargent DF, Luger K, Maeder AW, Richmond TJ. Solvent Mediated Interactions in the Structure of the Nucleosome Core Particle at 1.9 Å Resolution. J Mol Biol. 2002;319: 1097–1113. 12079350 10.1016/S0022-2836(02)00386-8

[5] Schalch T, Duda S, Sargent DF, Richmond TJ. X-ray structure of a tetranucleosome and its implications for the chromatin fibre. Nature. 2005;436: 138–141. 16001076 10.1038/nature03686

[6] Vasudevan D, Chua EYD, Davey CA. Crystal Structures of Nucleosome Core Particles Containing the “601” Strong Positioning Sequence. J Mol Biol. 2010;403: 1–10. 10.1016/j.jmb.2010.08.039 20800598

[7] Tachiwana H, Kagawa W, Shiga T, Osakabe A, Miya Y, Saito K, et al Crystal structure of the human centromeric nucleosome containing CENP-A. Nature. 2011;476: 232–235. 10.1038/nature10258 21743476

[8] Workman JL. Nucleosome displacement in transcription. Genes Dev. 2006;20: 2009–2017. 16882978 10.1101/gad.1435706

[9] Beshnova DA, Cherstvy AG, Vainshtein Y, Teif VB. Regulation of the Nucleosome Repeat Length In Vivo by the DNA Sequence, Protein Concentrations and Long-Range Interactions. PLoS Comput Biol. 2014;10: e1003698 10.1371/journal.pcbi.1003698 24992723 PMC4081033

[10] Hansen JC. CONFORMATIONAL DYNAMICS OF THE CHROMATIN FIBER IN SOLUTION: Determinants, Mechanisms, and Functions. Annu Rev Biophys Biomol Struct. 2002;31: 361–392. 11988475 10.1146/annurev.biophys.31.101101.140858

[11] Luger K, Dechassa ML, Tremethick DJ. New insights into nucleosome and chromatin structure: an ordered state or a disordered affair? Nat Rev Mol Cell Biol. 2012;13: 436–447. 10.1038/nrm3382 22722606 PMC3408961

[12] Polach KJ, Widom J. Mechanism of Protein Access to Specific DNA Sequences in Chromatin: A Dynamic Equilibrium Model for Gene Regulation. J Mol Biol. 1995;254: 130–149. 7490738 10.1006/jmbi.1995.0606

[13] Li G, Widom J. Nucleosomes facilitate their own invasion. Nat Struct Mol Biol. 2004;11: 763–769. 15258568 10.1038/nsmb801

[14] Park Y-J, Dyer PN, Tremethick DJ, Luger K. A New Fluorescence Resonance Energy Transfer Approach Demonstrates That the Histone Variant H2AZ Stabilizes the Histone Octamer within the Nucleosome. J Biol Chem. 2004;279: 24274–24282. 15020582 10.1074/jbc.M313152200

[15] Gansen A, Hauger F, Tóth K, Langowski J. Single-pair fluorescence resonance energy transfer of nucleosomes in free diffusion: Optimizing stability and resolution of subpopulations. Anal Biochem. 2007;368: 193–204. 17553453 10.1016/j.ab.2007.04.047

[16] Koopmans WJA, Brehm A, Logie C, Schmidt T, van Noort J. Single-Pair FRET Microscopy Reveals Mononucleosome Dynamics. J Fluoresc. 2007;17: 785–795. 17609864 10.1007/s10895-007-0218-9 PMC2064943

[17] Tims HS, Gurunathan K, Levitus M, Widom J. Dynamics of Nucleosome Invasion by DNA Binding Proteins. J Mol Biol. 2011;411: 430–448. 10.1016/j.jmb.2011.05.044 21669206 PMC3164294

[18] Cui Y, Bustamante C. Pulling a single chromatin fiber reveals the forces that maintain its higher-order structure. Proc Natl Acad Sci USA. 2000;97: 127–132. 10618382 10.1073/pnas.97.1.127 PMC26627

[19] Bennink ML, Leuba SH, Leno GH, Zlatanova J, de Grooth BG, Greve J. Unfolding individual nucleosomes by stretching single chromatin fibers with optical tweezers. Nat Struct Mol Biol. 2001;8: 606–610. 10.1038/89646 11427891

[20] Brower-Toland BD, Smith CL, Yeh RC, Lis JT, Peterson CL, Wang MD. Mechanical disruption of individual nucleosomes reveals a reversible multistage release of DNA. Proc Natl Acad Sci USA. 2002;99: 1960–1965. 11854495 10.1073/pnas.022638399 PMC122302

[21] Brower-Toland B, Wacker DA, Fulbright RM, Lis JT, Kraus WL, Wang MD. Specific Contributions of Histone Tails and their Acetylation to the Mechanical Stability of Nucleosomes. J Mol Biol. 2005;346: 135–146. 15663933 10.1016/j.jmb.2004.11.056

[22] Gemmen GJ, Sim R, Haushalter KA, Ke PC, Kadonaga JT, Smith DE. Forced Unraveling of Nucleosomes Assembled on Heterogeneous DNA Using Core Histones, NAP-1, and ACF. J Mol Biol. 2005;351: 89–99. 16002089 10.1016/j.jmb.2005.05.058

[23] Hall MA, Shundrovsky A, Bai L, Fulbright RM, Lis JT, Wang MD. High-resolution dynamic mapping of histone-DNA interactions in a nucleosome. Nat Struct Mol Biol. 2009;16: 124–129. 10.1038/nsmb.1526 19136959 PMC2635915

[24] Choy JS, Lee T-H. Structural dynamics of nucleosomes at single-molecule resolution. Trends Biochem Sci. 2012;37: 425–435. 10.1016/j.tibs.2012.06.006 22831768 PMC3669752

[25] Ettig R, Kepper N, Stehr R, Wedemann G, Rippe K. Dissecting DNA-Histone Interactions in the Nucleosome by Molecular Dynamics Simulations of DNA Unwrapping. Biophys J. 2011;101: 1999–2008. 10.1016/j.bpj.2011.07.057 22004754 PMC3192959

[26] Hyeon C, Thirumalai D. Capturing the essence of folding and functions of biomolecules using coarse-grained models. Nat Commun. 2011;2: 487 10.1038/ncomms1481 21952221

[27] Takada S. Coarse-grained molecular simulations of large biomolecules. Curr Opin Struct Biol. 2012;22: 130–137. 10.1016/j.sbi.2012.01.010 22365574

[28] Sun J, Zhang Q, Schlick T. Electrostatic mechanism of nucleosomal array folding revealed by computer simulation. Proc Natl Acad Sci USA. 2005;102: 8180–8185. 15919827 10.1073/pnas.0408867102 PMC1140479

[29] Sharma S, Ding F, Dokholyan NV. Multiscale Modeling of Nucleosome Dynamics. Biophys J. 2007;92: 1457–1470. 17142268 10.1529/biophysj.106.094805 PMC1796817

[30] Arya G, Schlick T. A Tale of Tails: How Histone Tails Mediate Chromatin Compaction in Different Salt and Linker Histone Environments. J Phys Chem A. 2009;113: 4045–4059. 10.1021/jp810375d 19298048 PMC2693032

[31] Wocjan T, Klenin K, Langowski J. Brownian Dynamics Simulation of DNA Unrolling from the Nucleosome†. J Phys Chem B. 2009;113: 2639–2646. 19708203 10.1021/jp806137e

[32] Cao Q, Zuo C, Ma Y, Li L, Zhang Z. Interaction of double-stranded DNA with a nanosphere: a coarse-grained molecular dynamics simulation study. Soft Matter. 2011;7: 506.

[33] Kepper N, Ettig R, Stehr R, Marnach S, Wedemann G, Rippe K. Force spectroscopy of chromatin fibers: Extracting energetics and structural information from Monte Carlo simulations. Biopolymers. 2011;95: 435–447. 10.1002/bip.21598 21294108

[34] Dobrovolskaia IV, Arya G. Dynamics of Forced Nucleosome Unraveling and Role of Nonuniform Histone-DNA Interactions. Biophys J. 2012;103: 989–998. 23009848 10.1016/j.bpj.2012.07.043 PMC3433614

[35] Voltz K, Trylska J, Calimet N, Smith JC, Langowski J. Unwrapping of Nucleosomal DNA Ends: A Multiscale Molecular Dynamics Study. Biophys J. 2012;102: 849–858. 10.1016/j.bpj.2011.11.4028 22385856 PMC3283802

[36] Fan Y, Korolev N, Lyubartsev AP, Nordenskiöld L. An Advanced Coarse-Grained Nucleosome Core Particle Model for Computer Simulations of Nucleosome-Nucleosome Interactions under Varying Ionic Conditions. PLoS ONE. 2013;8: e54228 10.1371/journal.pone.0054228 23418426 PMC3572162

[37] Fathizadeh A, Besya AB, Ejtehadi MR, Schiessel H. Rigid-body molecular dynamics of DNA inside a nucleosome. Eur Phys J E. 2013;36: 1–10. 23475204 10.1140/epje/i2013-13021-4

[38] Clementi C, Nymeyer H, Onuchic JN. Topological and energetic factors: what determines the structural details of the transition state ensemble and “en-route” intermediates for protein folding? an investigation for small globular proteins. J Mol Biol. 2000;298: 937–953. 10801360 10.1006/jmbi.2000.3693

[39] Koga N, Takada S. Roles of native topology and chain-length scaling in protein folding: A simulation study with a Gō-like model. J Mol Biol. 2001;313: 171–180. 11601854 10.1006/jmbi.2001.5037

[40] Knotts TA IV, Rathore N, Schwartz DC, de Pablo JJ. A coarse grain model for DNA. J Chem Phys. 2007;126: 084901 17343470 10.1063/1.2431804

[41] Sambriski EJ, Schwartz DC, de Pablo JJ. A Mesoscale Model of DNA and Its Renaturation. Biophys J. 2009;96: 1675–1690. 10.1016/j.bpj.2008.09.061 19254530 PMC2717267

[42] Kenzaki H, Koga N, Hori N, Kanada R, Li W, Okazaki K, et al CafeMol: A Coarse-Grained Biomolecular Simulator for Simulating Proteins at Work. J Chem Theory Comput. 2011;7: 1979–1989. 26596457 10.1021/ct2001045

[43] Savelyev A, Papoian GA. Chemically accurate coarse graining of double-stranded DNA. Proc Natl Acad Sci USA. 2010;107: 20340–20345. 10.1073/pnas.1001163107 21059937 PMC2996671

[44] Terakawa T, Kenzaki H, Takada S. p53 Searches on DNA by Rotation-Uncoupled Sliding at C-Terminal Tails and Restricted Hopping of Core Domains. J Am Chem Soc. 2012;134: 14555–14562. 10.1021/ja305369u 22880817

[45] Onuchic JN, Luthey-Schulten Z, Wolynes PG. THEORY OF PROTEIN FOLDING: The Energy Landscape Perspective. Annu Rev Phys Chem. 1997;48: 545–600. 9348663 10.1146/annurev.physchem.48.1.545

[46] Terakawa T, Takada S. Multiscale Ensemble Modeling of Intrinsically Disordered Proteins: p53 N-Terminal Domain. Biophys J. 2011;101: 1450–1458. 10.1016/j.bpj.2011.08.003 21943426 PMC3177054

[47] Mihardja S, Spakowitz AJ, Zhang Y, Bustamante C. Effect of force on mononucleosomal dynamics. Proc Natl Acad Sci USA. 2006;103: 15871–15876. 17043216 10.1073/pnas.0607526103 PMC1635095

[48] Sheinin MY, Li M, Soltani M, Luger K, Wang MD. Torque modulates nucleosome stability and facilitates H2A/H2B dimer loss. Nat Commun. 2013;4. 10.1038/ncomms3579 PMC3848035 24113677

[49] Freeman GS, Lequieu JP, Hinckley DM, Whitmer JK, de Pablo JJ. DNA Shape Dominates Sequence Affinity in Nucleosome Formation. Phys Rev Lett. 2014;113: 168101 25361282 10.1103/PhysRevLett.113.168101

