# Supplementary material for: Partial Unwrapping and Histone Tail Dynamics in Nucleosome Revealed by Coarse-Grained Molecular Simulations
Source: PLoS Comput Biol. 2015 Aug 11;11(8):e1004443. doi: 10.1371/journal.pcbi.1004443 (PMC4532510; doi:10.1371/journal.pcbi.1004443)
Supplement: S1 Table — All but εgopro−dna values used are the standard (default) values in CafeMol. (DOCX) [file pcbi.1004443.s001.docx]

| Parameter | Value |
| --- | --- |
| $\boldsymbol{\varepsilon}_{\boldsymbol{0}}^{\boldsymbol{pro}}$ | $1.0 kcal\cdot mol^{-1}$ |
| $\mathbf{k}_{\mathbf{bd}}^{\boldsymbol{pro}}$ | $100\varepsilon_{0}^{pro}$ |
| $\mathbf{k}_{\mathbf{ba}}^{\boldsymbol{pro}}$ | $20\varepsilon_{0}^{pro}$ |
| $\mathbf{k}_{\mathbf{dih1}}^{\boldsymbol{pro}}$ | $1\varepsilon_{0}^{pro}$ |
| $\mathbf{k}_{\mathbf{dih3}}^{\boldsymbol{pro}}$ | $0.5\varepsilon_{0}^{pro}$ |
| $\boldsymbol{\varepsilon}_{\mathbf{go}}^{\boldsymbol{pro}}$ | $0.3\varepsilon_{0}^{pro}$ |
| $\boldsymbol{\varepsilon}_{\mathbf{ev}}^{\boldsymbol{pro}}$ | ${0.2\varepsilon}_{0}^{pro}$ |
| $\boldsymbol{\sigma}^{\boldsymbol{pro}}$ | $6 Å$ |
| $\boldsymbol{\varepsilon}_{\mathbf{0}}^{\boldsymbol{dna}}$ | $0.769856 kcal\cdot mol^{-1}$ |
| $\mathbf{k}_{\mathbf{bd1}}^{\boldsymbol{dna}}$ | $\varepsilon_{0}^{dna}$ |
| $\mathbf{k}_{\mathbf{bd2}}^{\boldsymbol{dna}}$ | $100\varepsilon_{0}^{dna}$ |
| $\mathbf{k}_{\mathbf{ba}}^{\boldsymbol{dna}}$ | $1400\varepsilon_{0}^{dna}$ |
| $\mathbf{k}_{\boldsymbol{\phi}}^{\boldsymbol{dna}}$ | ${28\varepsilon}_{0}^{dna}$ |
| $\boldsymbol{\varepsilon}_{\mathbf{st}}^{\boldsymbol{dna}}$ | $\varepsilon_{0}^{dna}$ |
| $\boldsymbol{\varepsilon}_{\mathbf{AT}}^{\boldsymbol{dna}}$ | 2.000$\varepsilon_{0}^{dna}$ |
| $\boldsymbol{\varepsilon}_{\mathbf{CG}}^{\boldsymbol{dna}}$ | 2.532$\varepsilon_{0}^{dna}$ |
| $\boldsymbol{\varepsilon}_{\mathbf{ev}}^{\boldsymbol{dna}}$ | $\varepsilon_{0}^{dna}$ |
| $\boldsymbol{\varepsilon}_{\mathbf{s}}$ | $\varepsilon_{0}^{dna}$ |
| $\boldsymbol{\sigma}_{\boldsymbol{AT}}^{\boldsymbol{dna}}$ | $2.9002 Å$ |
| $\boldsymbol{\sigma}_{\boldsymbol{CG}}^{\boldsymbol{dna}}$ | $2.8694 Å$ |
| $\boldsymbol{\sigma}^{\boldsymbol{dna}}\mathbf{(mismatch base pair)}$ | ${1.00\cdot2}^{-1/6} Å$ |
| $\boldsymbol{\sigma}^{\boldsymbol{dna}}\mathbf{(otherwise)}$ | ${6.86\cdot2}^{-1/6} Å$ |
| $\boldsymbol{\varepsilon}$ | $78$ |
| $\boldsymbol{\lambda}_{\mathbf{D}}$ | $\left[ \frac{\epsilon_{0}\epsilon k_{B}T}{2N_{A}e^{2}I} \right]^{1/2}Å$ |
| $\mathbf{T}$ | $300K$ |
| $\mathbf{I}$ | ionic strength |
| $\boldsymbol{\alpha}^{\boldsymbol{-}\boldsymbol{1}}$ | $5.333 Å$ |
| $\mathbf{r}_{\mathbf{s}}$ | $13.38 Å$ |
| $\boldsymbol{\varepsilon}_{\mathbf{go}}^{\boldsymbol{pro}\boldsymbol{-dna}}$ | control parameter |
| $\boldsymbol{\varepsilon}_{\mathbf{ev}}^{\boldsymbol{pro}\boldsymbol{-dna}}$ | $\varepsilon_{\mathrm{ev}}^{pro}$ |
| $\boldsymbol{\sigma}^{\boldsymbol{pro}\boldsymbol{-dna}}$ | $\sigma^{\mathrm{pro}}$ |

**S1 Table. Parameters of Coarse-grained protein and DNA models.**
