# Supplementary figures and images for: Partial Unwrapping and Histone Tail Dynamics in Nucleosome Revealed by Coarse-Grained Molecular Simulations
Source: PLoS Comput Biol. 2015 Aug 11;11(8):e1004443. doi: 10.1371/journal.pcbi.1004443 (PMC4532510; doi:10.1371/journal.pcbi.1004443)

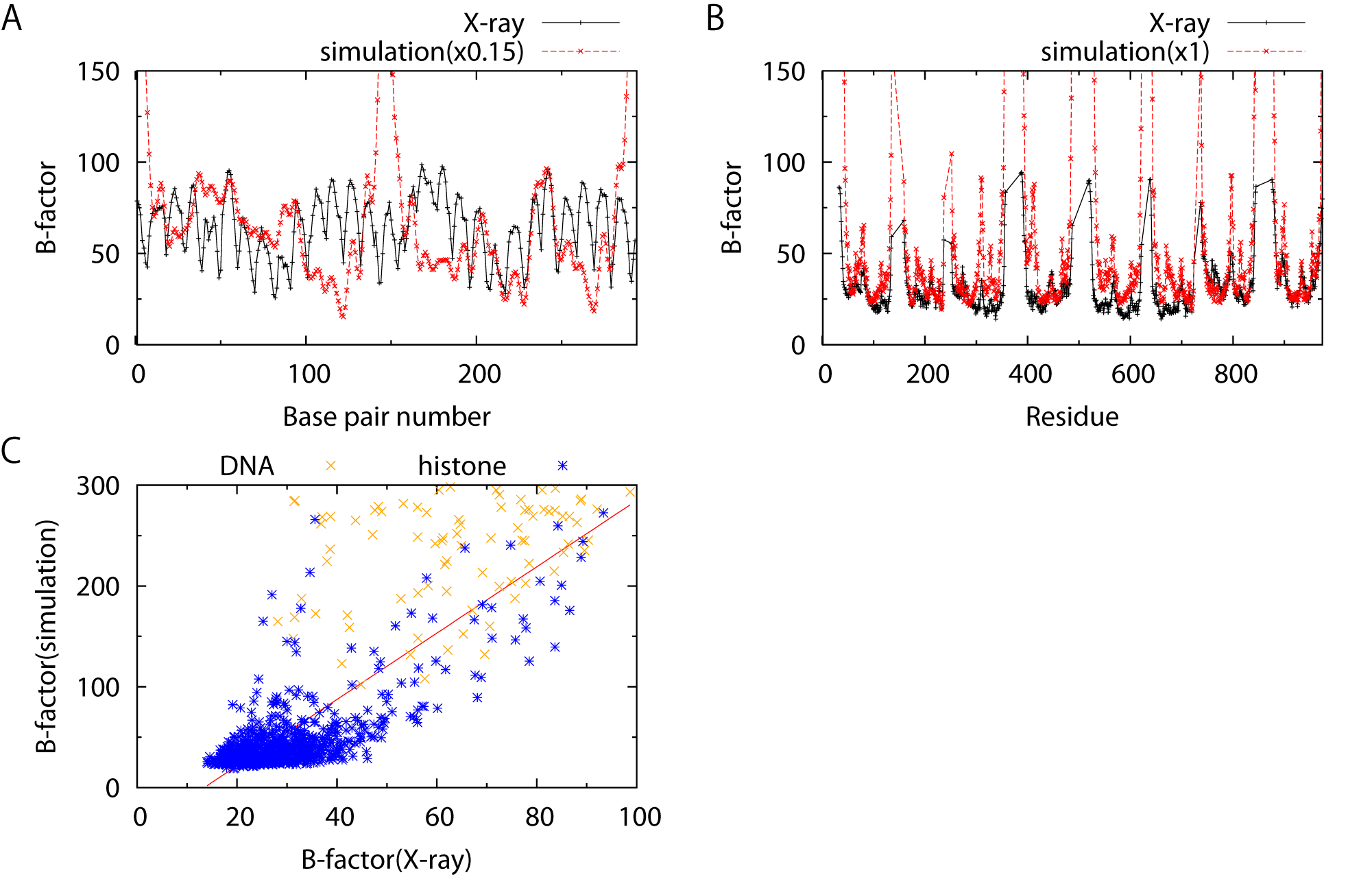

Supplement: S1 Fig — B-factor of the X-ray crystallography (black) and that calculated from simulations (red) for (A) DNA and histones (B). For DNA, B-factor is plotted for O5’ atom in the X-ray crystallographic data and for the sugar bead in the coarse-grained model. For histones, Cα atom is used for B-factor, except for disordered regions of histone tails in the X-ray crystal structure. For simulation, salt concentration is NaCl 100 mM and εgopro−dna=0.8εgopro. The root-mean-square fluctuations of DNA are about 3–5 Å and those of histone core are about 1–2 Å; DNA beads have larger fluctuations in the simulations. (C) The correlation plot between experimental and calculated B-factors. The correlation coefficient was 0.80 for the entire system when the calculated B-factor larger than 300 were excluded. We note that the agreement for DNA is lower due to many possible reasons. The experimental B-factor is affected by crystallographic environment and global rotation, while the computation B-factor here does not take into account the crystallographic environment. Even among experimental data, B-factors in nucleosomes are largely different: Comparison of B-factors in several nucleosome crystallographic data revealed that they are little conserved overall and only the conserved feature is the periodicity of 10 bp. Importantly, the simulated B-factor reproduced this feature. (TIF) [file pcbi.1004443.s002.tif]

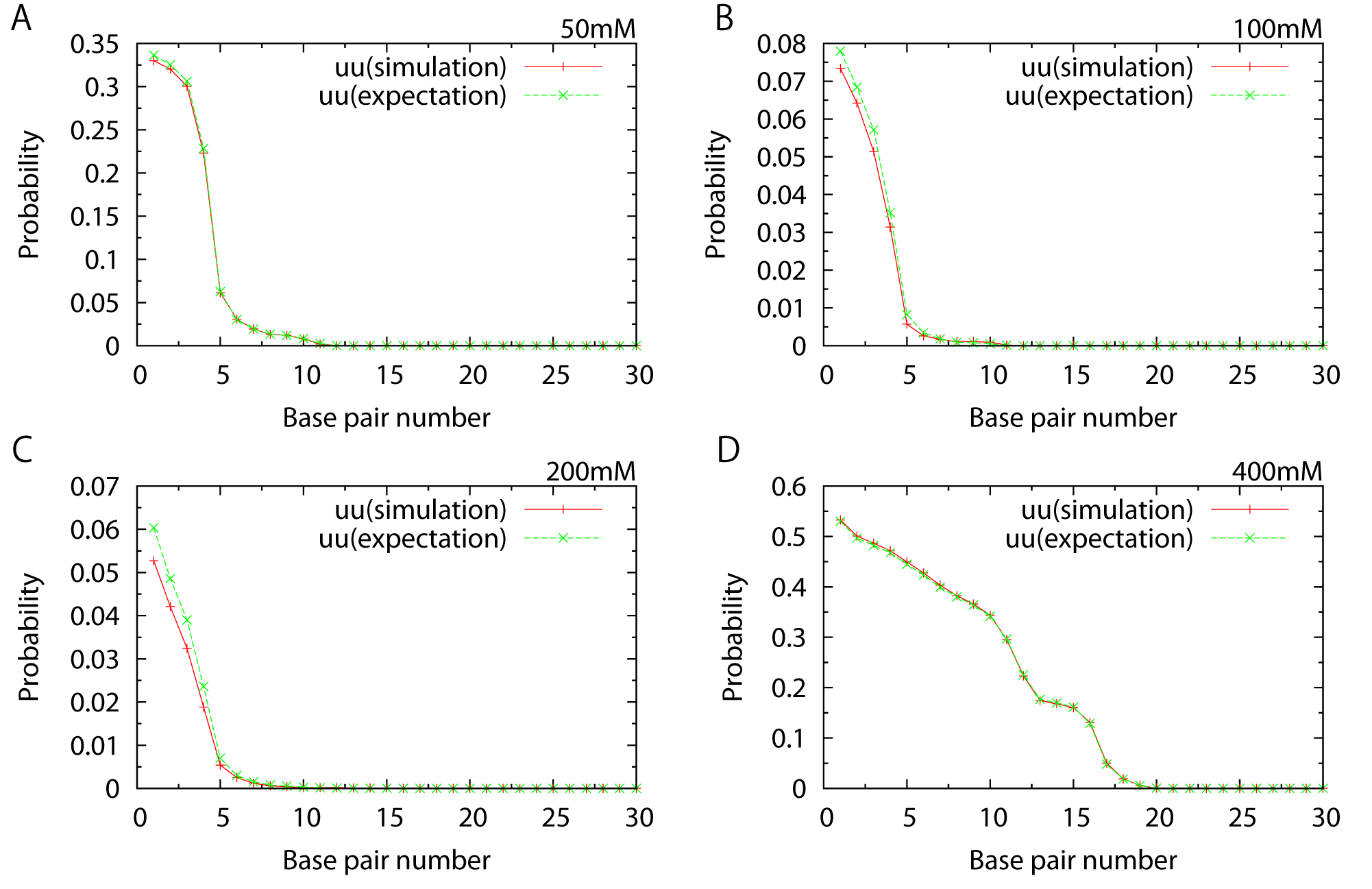

Supplement: S2 Fig — Firstly, for a given snapshot, i-th base pair from terminus was assigned as either wrapped or unwrapped. The probability that i-th base pairs in both ends are in the unwrapped states were calculated (red curve designated as uu(simulations)). Under the assumption of independence of unwrapping of two ends, we estimated the expectation value that both ends are unwrapped, i.e., the product of probabilities of unwrapping of each ends (green curve designated as uu(expectation)). Results with the salt concentrations, NaCl 50 mM (A), 100 mM (B), 200 mM (C), and 400 mM (D). The correlation between partial unwrapping of the left and right end of DNA is insignificant. (TIF) [file pcbi.1004443.s003.tif]

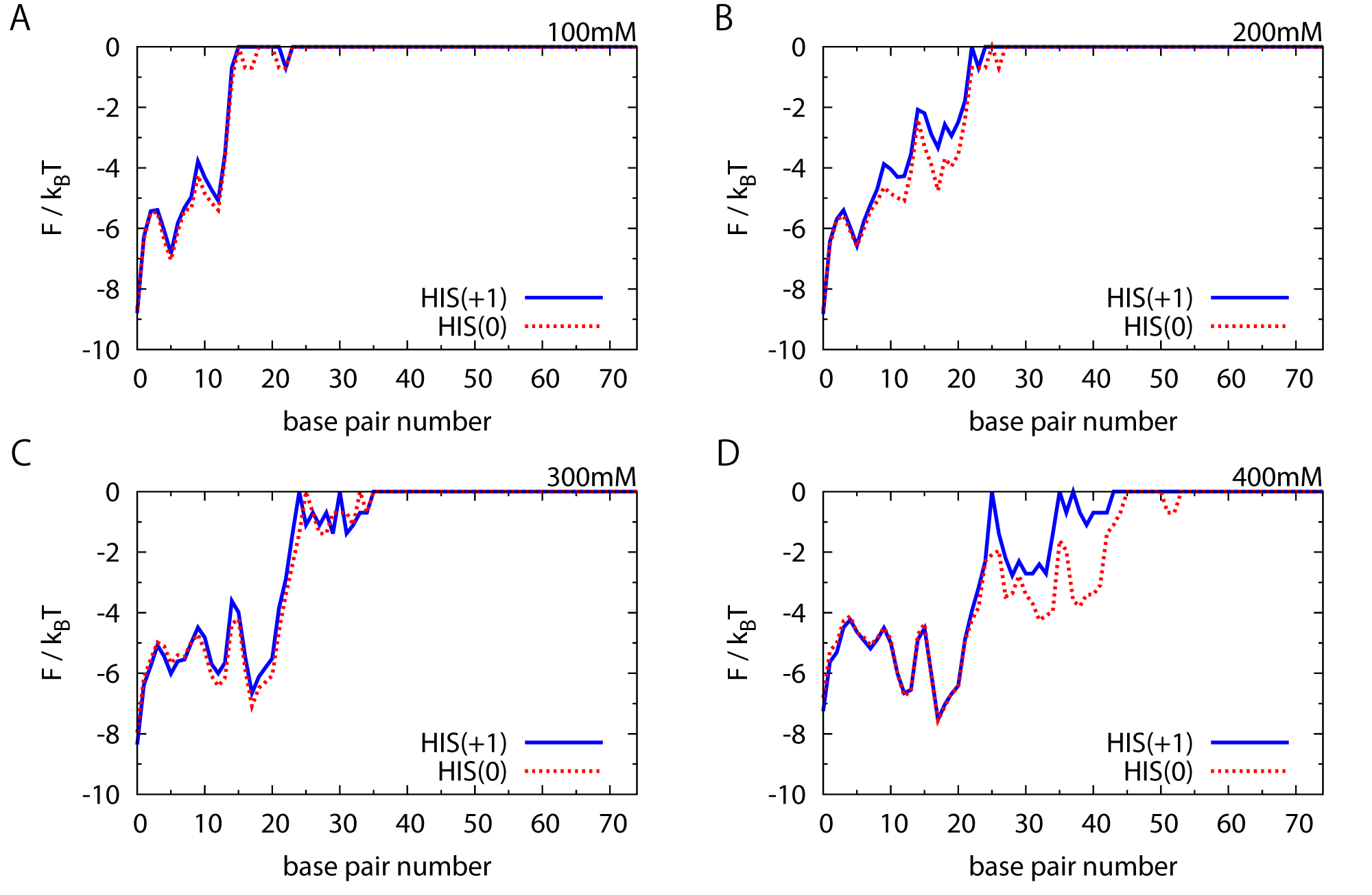

Supplement: S3 Fig — Free energy profiles for the left end unwrapped state with histidine charge equal to +1 (blue) and zero (red) at NaCl 100 mM (A), at 200 mM (B), at 300 mM (C), and at 400 mM (D). Histidine residues in histone proteins are relatively few; histone octamer contains 18 histidine residues, whereas 106 arginine residues and 114 lysine residues. (TIF) [file pcbi.1004443.s004.tif]

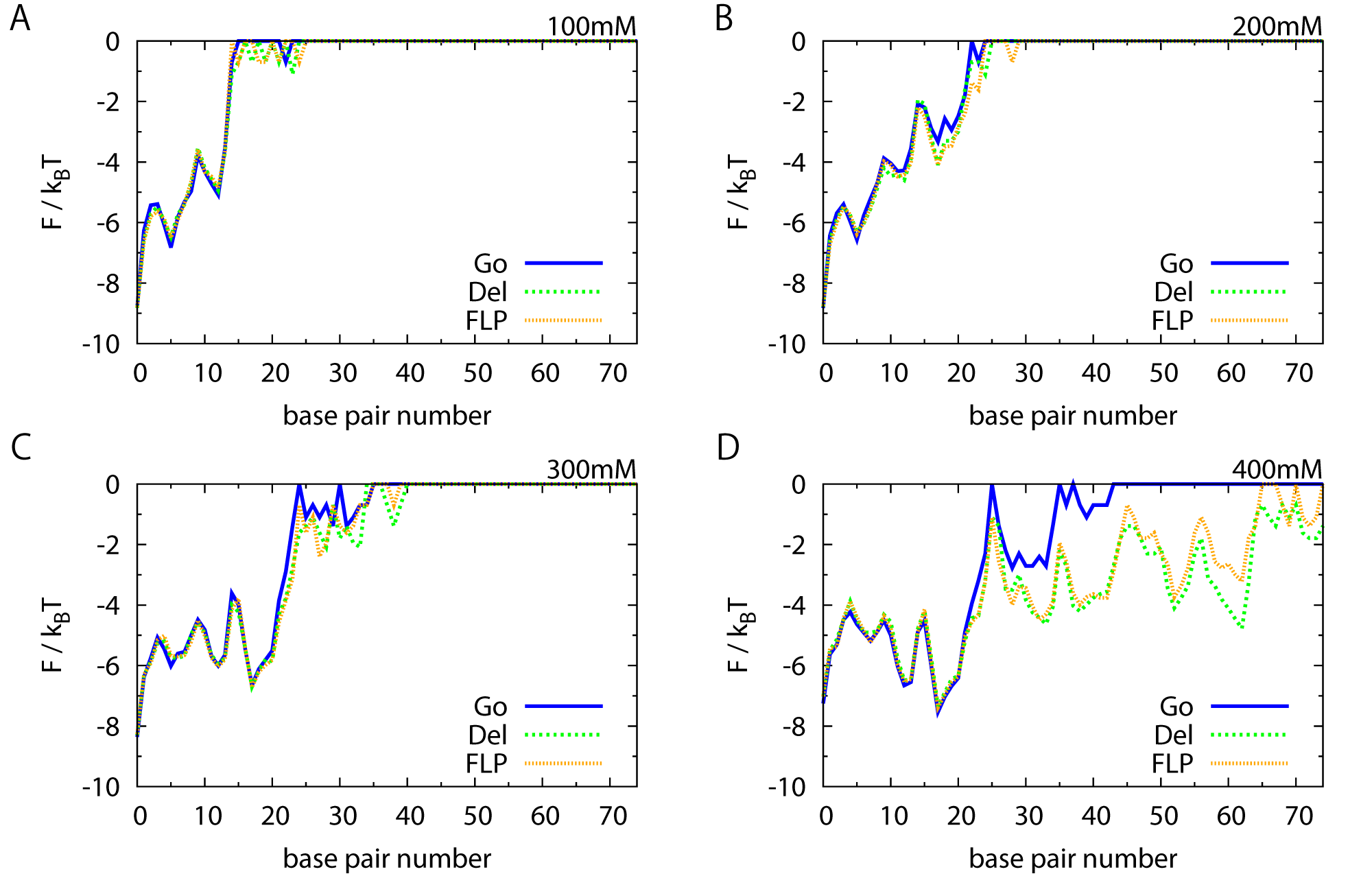

Supplement: S4 Fig — Histone tails are largely disordered; residues 1–32 of chain H3, 1–30 of H3’, 1–23 of H4, 1–15 of H4’, 1–14 and 121–128 of H2A, 1–12 and 122–128 of H2A’, 4–24 of H2B, and 4–26 of H2B’ are disordered. We tested different modeling for these disordered regions. “Go” (blue) means that we used the Go interaction based on the modeled structure in 1KX5.pdb (one that was used in the main text). “Del” (green) means that the Go interaction was deleted except for bond length term. “FLP” (orange) is the case that we deleted Go interaction and applied the statistical potential. Results with the salt concentrations, NaCl 100 mM (A), 200 mM (B), 300 mM (C), and 400 mM (D). (TIF) [file pcbi.1004443.s005.tif]
